# Gender-Affirming Surgery for Transgender and Gender Diverse Medicare Beneficiaries

**DOI:** 10.1001/jamanetworkopen.2025.8072

**Published:** 2025-05-01

**Authors:** Em Balkan, Gray Babbs, David J. Meyers, Patrick J. A. Kelly, Kim Yee, David R. Pletta, Theresa I. Shireman, Ash B. Alpert, Kellan E. Baker, Jaclyn M. W. Hughto

**Affiliations:** 1 Department of Health Services, Policy & Practice, Brown University School of Public Health, Providence, Rhode Island; 2 The Center for Gerontology and Healthcare Research, Brown University, Providence, Rhode Island; 3 Department of Behavioral and Social Sciences, Brown University School of Public Health, Providence, Rhode Island; 4 Oregon Health and Science University–Portland State University School of Public Health, Portland; 5 Yale Cancer Center, New Haven, Connecticut; 6 Yale School of Medicine, New Haven, Connecticut; 7 The Institutes for Health Research & Policy at Whitman-Walker, Washington, DC; 8 Johns Hopkins Bloomberg School of Public Health, Baltimore, Maryland; 9 Department of Epidemiology, Brown University School of Public Health, Providence, Rhode Island; 10 Center for Health Promotion and Health Equity, Brown University, Providence, Rhode Island

## Abstract

**Importance:**

Medicare covers gender-affirming surgical procedures on a case-by-case basis. The proportion of Medicare beneficiaries who receive gender-affirming surgical procedures is unknown.

**Objective:**

To examine the frequency and trends of gender-affirming surgical procedures for Medicare beneficiaries.

**Design, Setting, and Participants:**

This cross-sectional study used national Medicare data to analyze transgender and gender diverse beneficiaries’ use of gender-affirming surgical procedures from January 1, 2016, to February 29, 2020, as well as use of the same surgical procedures by beneficiaries not identified as transgender. Transgender Medicare beneficiaries were identified using a claims-based algorithm; nontransgender individuals were selected using propensity score matching. Analyses were conducted from November 2022 through October 2024.

**Exposures:**

Race and ethnicity, Medicare-Medicaid dual status, age, original reason for Medicare entitlement, geographic area, and chronic conditions.

**Main Outcomes and Measures:**

Descriptive analyses and generalized estimating equations were used to assess gender-affirming surgical procedures and beneficiary characteristics associated with surgical procedures.

**Results:**

This study included transgender individuals (mean [SD] age, 60.0 [18.6] years; 43.2% transfeminine individuals, 33.0% transmasculine individuals, and 23.9% individuals with unclassified gender) and individuals (mean [SD] age, 62.7 [21.0] years; 55.5% women and 44.5% men) not identified as transgender. Of 142 703 total person-years, there were 2156 instances of transgender individuals having at least 1 gender-affirming surgical procedure in the calendar year. The rate of individuals receiving at least 1 surgical procedure decreased from between 2.1% and 2.2% in 2016 and 2017 to 1.4% in 2018 and 2019. There was substantial variability in surgical procedures across beneficiary characteristics. Transgender beneficiaries who underwent surgery were younger (31-40 years vs 61-65 years: adjusted odds ratio [AOR], 1.91 [95% CI, 1.55-2.34]) and had higher enrollment in Medicaid (AOR, 1.14 [95% CI, 1.02-1.26]), higher rates of chronic conditions (≥10 conditions vs 0: AOR, 2.10 [95% CI, 1.79-2.46]), and higher proportions of residents on the West Coast (Alaska, Idaho, Oregon, and Washington) vs the Northeast (Connecticut, Maine, Massachusetts, New Hampshire, Rhode Island, and Vermont) (AOR, 1.30 [95% CI, 1.05-1.61]) compared with those who did not undergo surgery. Geographically, the largest difference was observed when comparing the Northeast with the Southeast (Alabama, Florida, Georgia, Kentucky, Mississippi, North Carolina, South Carolina, and Tennessee) (AOR, 0.70 [95% CI, 0.58-0.86]; *P* < .001) and other parts of the South (Arkansas, Louisiana, New Mexico, Oklahoma, and Texas) (AOR, 0.56 [95% CI, 0.44-0.71]; *P* < .001). There was no significant difference in receipt of surgical procedures across regions for those not identified as transgender.

**Conclusions and Relevance:**

This cross-sectional study of the Medicare program found that gender-affirming surgical procedures were very rare, particularly for those who were older, were not dually enrolled in Medicare and Medicaid, and who resided in the South. Although there were substantive regional differences in receipt of gender-affirming surgical procedures among transgender Medicare beneficiaries, beneficiaries not identified as transgender did not experience regional differences. Further study is warranted to examine barriers to accessing gender-affirming surgery for transgender and gender diverse beneficiaries.

## Key Points

**Question:**

What were the frequency and trends of gender-affirming surgical procedures for transgender and gender diverse Medicare beneficiaries from 2016 to 2020?

**Findings:**

In this cross-sectional study of 35 737 Medicare beneficiaries identified as transgender, gender-affirming surgical procedures were very rare, and the rate of surgery receipt decreased from between 2.1% and 2.2% in 2016 and 2017 to 1.4% in 2018 and 2019. There were also significant differences in the frequency of gender-affirming surgical procedures by beneficiary characteristics.

**Meaning:**

This study suggests that few transgender Medicare beneficiaries undergo gender-affirming surgery, and further study is warranted to understand any barriers to accessing care.

## Teaser

This cross-sectional study examines the frequency and trends of gender-affirming surgical procedures for transgender and gender diverse Medicare beneficiaries from 2016 to 2020.

## Introduction

Transgender and gender diverse (TGD)^1^ individuals may seek gender-affirming surgery (eg, mastectomy and phalloplasty) to align their bodily features with their gender identity. Gender-affirming surgery is cost-effective^2,3^ and associated with improved mental health outcomes, including lower rates of suicidal ideation, depression, and anxiety.^4,5,6^ Given its strong association with these benefits, gender-affirming surgery is considered medically necessary care by major medical organizations in the US.^7,8^

Although it is difficult to estimate, survey-based studies estimate the lifetime receipt of gender-affirming surgery for TGD people to be 16% to 40%.^9,10,11^ Moreover, studies of insured and self-paying TGD people point to an increase in gender-affirming surgical procedures over time.^11,12,13,14,15,16,17^ Prior studies suggest that this increase is at least partly due to expanded coverage by the Centers for Medicare & Medicaid Services (CMS).^18^ In 2014, CMS issued a notice stating that regional Medicare Administrative Contractors could determine coverage for gender-affirming surgery on a case-by-case basis.^19^ Medicare Administrative Contractors are private health care insurers that are awarded a geographic jurisdiction to process medical claims for Medicare fee-for-service beneficiaries.^19^

Although prior research has examined gender-affirming surgical procedures among all insurers or Medicaid,^6,11,13,14,16,17,20^ to our knowledge, no study has been conducted exclusively among Medicare beneficiaries. Research with this subpopulation is needed to examine any barriers to care for older adults and those with disabilities and, if relevant, identify possible points of intervention. To fill gaps in the literature, our study had 2 primary research objectives. First, we examined the national population proportion of beneficiaries undergoing gender-affirming surgery within the Medicare program over time across different demographic groups. Second, we examined gender-affirming surgical procedures among TGD Medicare beneficiaries by beneficiary characteristics, including race and ethnicity, age, geography, dual Medicare-Medicaid enrollment, original reason for Medicare entitlement, and number of chronic conditions. We compared the rate of gender-affirming surgical procedures among TGD beneficiaries with a group of individuals who received the same surgical procedures but were not identified as TGD (hereafter referred to as *non-TGD*) to identify any differences in care patterns.

## Methods

### Data Sources

Our primary data sources were Medicare Provider Analysis and Review files, Outpatient files, and claim- and line-level carrier files from January 1, 2016, to February 29, 2020, to identify beneficiaries within our sample who received gender-affirming surgery. We used the Medicare Provider Analysis and Review files to identify gender-affirming surgical procedures that occurred in inpatient settings and outpatient files to identify gender-affirming surgical procedures that occurred in outpatient settings.^21,22^ We used Master Beneficiary Summary Files to identify beneficiary characteristics.^23^ The Institutional Review Board of Brown University determined that this study was exempt. Informed consent requirements were waived due to the use of deidentified secondary data. This cross-sectional study followed the Strengthening the Reporting of Observational Studies in Epidemiology (STROBE) reporting guideline.

### Sample

We identified a TGD cohort from 100% of Medicare beneficiaries aged 18 years or older. TGD beneficiaries were identified using an algorithm that combines diagnosis, procedure, and prescription drug codes.^24,25^ A similar algorithm was validated with self-reported gender identity information in electronic health records and was found to have a high sensitivity (87%) and specificity (98%) in identifying TGD adults.^25^ Individuals identified as TGD were considered TGD for all observable time.

The non-TGD comparison group was selected from those not identified as TGD by our algorithm (ie, all other Medicare beneficiaries) who had at least 1 inpatient or outpatient claim and were aged 18 years or older. From this group, we selected a non-TGD cohort using propensity score matching on race and ethnicity, CMS region, dual Medicaid-Medicare enrollment status, original reason for Medicare entitlement, and age at the beginning of each year.^26^ This matching enabled us to select a non-TGD comparison group with similar demographic characteristics as the TGD cohort (ie, younger and more dually enrolled in Medicare and Medicaid compared with the general Medicare population) (eFigure in Supplement 1). For both the TGD and non-TGD comparison groups, we restricted the samples to those who were not enrolled in a Medicare Advantage plan for 6 or more months within a calendar year.

We identified over 850 gender-affirming surgery codes from our prior research and other studies using commercial insurance, all payer, and other data.^13,17,24^ We excluded procedures that were excluded from Medicare coverage (eg, facial feminization and liposuction), as stated in guidance published by a Medicare Administrative Contractor, reducing our list to 607 gender-affirming surgery codes (eTable 1 in Supplement 1).^27^ Gender-affirming surgical procedures were identified using *International Classification of Diseases, Ninth Revision*; *International Statistical Classification of Diseases and Related Health Problems, Tenth Revision* (*ICD-10*); and *Current Procedural Terminology* or Healthcare Common Procedure Coding System codes. For TGD beneficiaries, we did not consider surgical procedures that may have occurred due to a cancer diagnosis to be gender affirming (eTable 2 in Supplement 1). Although surgical procedures may be indicated for gender-affirming and cancer-related care,^28^ we excluded surgical procedures that may have been strictly related to cancer care to improve the algorithm’s specificity. We did this by removing claims that included a related cancer diagnosis.

### Covariates

All covariates were determined using Master Beneficiary Summary Files. Age was defined as the age of the individual at the start of the year. Age was categorized into 1 of 9 groups. Race and ethnicity were categorized from the files’ combined race and ethnicity variable as American Indian or Alaska Native, Asian or Other Pacific Islander, Black or African American, Hispanic, non-Hispanic White, unknown, or other. Medicare’s classification of “other” can be interpreted as a non-Hispanic race and ethnicity category other than those offered.^29^ We assessed beneficiaries’ race and ethnicity because observed differences in receipt of gender-affirming surgery by race and ethnicity may be associated with racism, including mechanisms of structural racism.^30,31,32^

We classified a beneficiary as dually enrolled each year they had both Medicare and Medicaid for at least 6 months. The original reason for Medicare entitlement was categorized as age, disability, end-stage kidney disease, or end-stage kidney disease and having a disability. CMS regions include states and US territories.^26^ Chronic conditions were identified using Chronic Conditions Data Warehouse (CCW) codes^33^ and Other Chronic or Potentially Disabling Conditions variables^34^ and were categorized as 0, 1, 2 to 5, 6 to 9, and 10 or more chronic conditions. In 2016, CCW codes were unavailable for 5 conditions: Parkinson disease, pneumonia, urologic cancer, dementia, and a combined mental health variable inclusive of depression and bipolar disorder. However, other mental health variables and an Alzheimer disease and related disease variable were available, and Parkinson disease, pneumonia, and urologic cancer were uncommon.

### Statistical Analysis

Analyses were conducted from November 2022 through October 2024. After identifying instances of gender-affirming surgery, we aggregated our data to the person-year level. Each beneficiary was represented in each year they had Medicare, with a binary variable indicating undergoing at least 1 gender-affirming surgical procedure or not. All comparisons used Pearson χ^2^ tests. We descriptively compared the characteristics of TGD beneficiaries who underwent gender-affirming surgery with the characteristics of those who did not. We then used logistic generalized estimating equations^35^ with an exchangeable correlation structure to estimate the odds of gender-affirming surgery from January 1, 2016, through February 29, 2020. All *P* values were from 2-sided tests and results were deemed statistically significant at *P* < .05. We included race and ethnicity, dual Medicaid-Medicare enrollment status, age group, reason for Medicare entitlement at enrollment, CMS region, and chronic conditions in our model. After using propensity score matching to create a non-TGD cohort, we flagged the same procedures and examined trends. All analyses were conducted using Stata/MP, version 18.0 (StataCorp LLC).

## Results

### Gender-Affirming Surgical Procedures Among the TGD Sample

**Figure 1.**
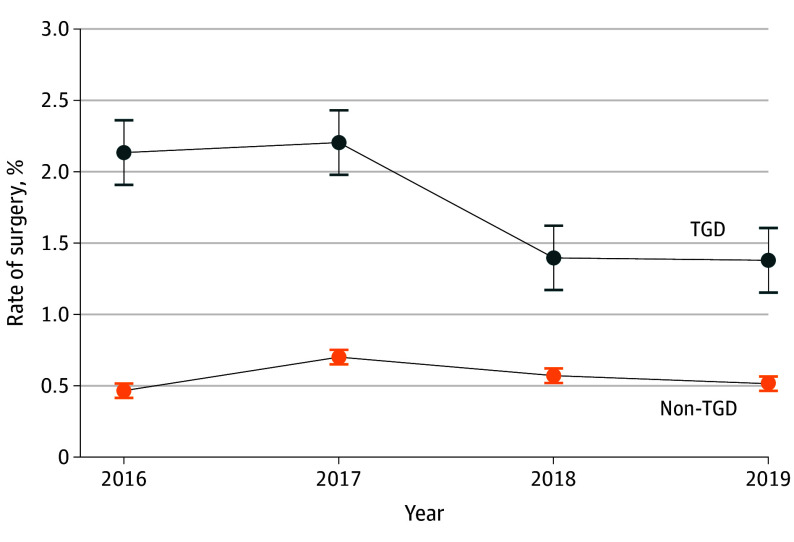
Proportion of Medicare Beneficiaries Receiving at Least 1 Gender-Affirming or Comparable Surgical Procedure by Transgender or Gender Diverse (TGD) Status, January 1, 2016, to December 31, 2019 Because the study period ended March 2020, the calendar year 2020 is excluded from the figure. Error bars indicate 95% CIs.

This study included 35 737 TGD individuals (mean [SD] age, 60.0 [18.6] years; 43.2% transfeminine individuals, 33.0% transmasculine individuals, and 23.9% individuals with an unclassified gender) and 101 361 non-TGD (mean [SD] age, 62.7 [21.0] years; 55.5% women and 44.5% men). Transgender and gender diverse beneficiaries who underwent gender-affirming surgery had higher rates of dual enrollment in Medicare and Medicaid (45.6% vs 34.2%; *P* < .001), were younger (aged ≤50 years: 44.3% vs 29.6%; *P* < .001), were less likely to be Medicare eligible due to age (30.1% vs 44.1%; *P* < .001), had higher rates of chronic conditions (no chronic conditions: 12.9% vs 16.7%; *P* < .001), had a higher proportion of individuals identified as Black or African American race (13.2% vs 11.3%; *P* = .01), and had higher proportions of individuals living in the Northeast (10.2% vs 8.0%; *P* < .001) or the West Coast (9.2% vs 5.9%; *P* < .001) compared with TGD beneficiaries who did not undergo surgery (all comparisons using Pearson χ^2^ tests) (Table 1).^29^ Our TGD sample included 142 703 beneficiary-year observations for 35 737 individuals from January 1, 2016, through February 29, 2020. Of these beneficiary-year observations, there were 2156 instances in which individuals had at least 1 gender-affirming surgery claim (Table 1).^29^ The rate of receipt of gender-affirming surgical procedures for TGD individuals was between 2.1% and 2.2% in 2016 and 2017 and decreased to 1.4% in 2018 and 2019 (Figure 1). The probability of receipt of at least 1 gender-affirming surgical procedure for TGD beneficiaries was 9.9 per 1000 person-years (95% CI, 8.0-11.8 per 1000 person-years) to 22.8 per 1000 person-years (95% CI, 19.4-26.3 per 1000 person-years) throughout the 10 CMS regions (eTable 3 in Supplement 1).

**Table 1.** Characteristics of Transgender and Gender Diverse Beneficiaries Who Did or Did Not Receive GAS at the Person-Year Level, January 2016 to February 2020

Characteristic	No. (%)		*P* value^a^
	No GAS (n = 140 547)	Received GAS (n = 2156)	
Medicare-Medicaid dual enrollment	48 062 (34.2)	983 (45.6)	<.001
Age, y			
18-30	13 206 (9.4)	254 (11.8)	<.001
31-40	14 330 (10.2)	369 (17.1)	
41-50	14 114 (10.0)	333 (15.4)	
51-55	8616 (6.1)	177 (8.2)	
56-60	8865 (6.3)	153 (7.1)	
61-65	13 642 (9.7)	186 (8.6)	
66-70	22 337 (15.9)	290 (13.5)	
71-75	16 670 (11.9)	182 (8.4)	
>75	28 767 (20.5)	212 (9.8)	
Race and ethnicity			
American Indian or Alaska Native	1109 (0.8)	17 (0.8)	.01
Asian or Other Pacific Islander	2669 (1.9)	35 (1.6)	
Black or African American	15 914 (11.3)	284 (13.2)	
Hispanic	8878 (6.3)	148 (6.9)	
Non-Hispanic White	107 974 (76.8)	1625 (75.4)	
Unknown	3019 (2.1)	42 (1.9)	
Other^b^	984 (0.7)	(0.2)	
Original reason for Medicare entitlement			
Age	62 011 (44.1)	648 (30.1)	<.001
Disability	76 619 (54.5)	1475 (68.4)	
ESKD or ESKD and disability	1917 (1.4)	33 (1.5)	
CMS region			
Region 1 (Connecticut, Maine, Massachusetts, New Hampshire, Rhode Island, Vermont)	11 215 (8.0)	219 (10.2)	<.001
Region 2 (New Jersey, New York, Puerto Rico, Virgin Islands)	13 914 (9.9)	189 (8.8)	
Region 3 (Delaware, District of Columbia, Maryland, Pennsylvania, Virginia, West Virginia)	14 437 (10.3)	216 (10.0)	
Region 4 (Alabama, Florida, Georgia, Kentucky, Mississippi, North Carolina, South Carolina, Tennessee)	24 393 (17.4)	292 (13.5)	
Region 5 (Illinois, Indiana, Michigan, Minnesota, Ohio, Wisconsin)	23 101 (16.4)	378 (17.5)	
Region 6 (Arkansas, Louisiana, New Mexico, Oklahoma, Texas)	13 238 (9.4)	129 (6.0)	
Region 7 (Iowa, Kansas, Missouri, Nebraska)	6923 (4.9)	105 (4.9)	
Region 8 (Colorado, Montana, North Dakota, South Dakota, Utah, Wyoming)	5370 (3.8)	76 (3.5)	
Region 9 (Arizona, California, Hawaii, Nevada, Pacific Territories)	19 536 (13.9)	351 (16.3)	
Region 10 (Alaska, Idaho, Oregon, Washington)	8288 (5.9)	198 (9.2)	
Other or living outside the 10 CMS regions	132 (0.1)	(0.1)	
Chronic conditions, No.			
0	23 421 (16.7)	278 (12.9)	<.001
1	7700 (5.5)	122 (5.7)	
2-5	39 467 (28.1)	631 (29.3)	
6-9	35 656 (25.4)	518 (24.0)	
≥10	34 303 (24.4)	607 (28.2)	

Abbreviation: CMS, Centers for Medicare & Medicaid Services; ESKD, end-stage kidney disease; GAS, gender-affirming surgery.

^a^ All variables are categorical or binary and are compared using the Pearson χ^2^ test.

^b^ Medicare’s classification of “other” can be interpreted as a non-Hispanic race and ethnicity other than those offered.^29^

**Figure 2.**
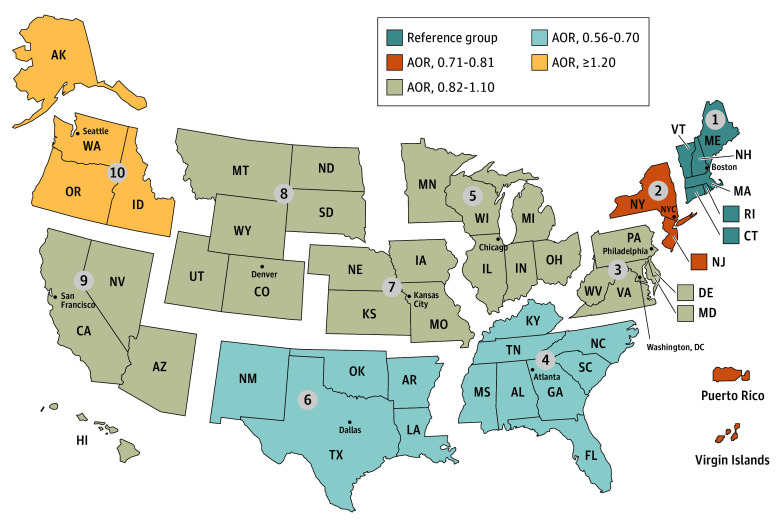
Significant Associations of Centers for Medicare & Medicaid Services Regions With Gender-Affirming Surgery, January 2016 to February 2020 Region 1: reference group; region 2: adjusted odds ratio (AOR), 0.79 (95% CI, 0.64-0.98); region 3: AOR, 0.82 (95% CI, 0.66-1.01); region 4: AOR, 0.70 (95% CI, 0.58-0.86); region 5: AOR, 0.88 (95% CI, 0.73-1.06); region 6: AOR, 0.56 (95% CI, 0.44-0.71); region 7: AOR, 0.82 (95% CI, 0.64-1.06); region 8: AOR, 0.82 (95% CI, 0.61-1.09); region 9: AOR, 1.04 (95% CI, 0.86-1.26); region 10: AOR, 1.30 (95% CI, 1.05-1.61).

In our adjusted analysis, we found significant variability in gender-affirming surgical procedures among TGD beneficiaries across our observed covariates (Table 2),^29^ including CMS region (Figure 2). We used CMS region 1 (the Northeast: Connecticut, Maine, Massachusetts, New Hampshire, Rhode Island, and Vermont) as our reference group. Previous studies indicated that a high proportion of gender-affirming surgical procedures occurred in the Northeast.^13,36^ Compared with those in CMS region 1, those residing in New Jersey, New York, Puerto Rico, and the Virgin Islands (region 2; AOR, 0.79 [95% CI, 0.64-0.98]; *P* = .03) had a lower adjusted odds of receiving gender-affirming surgery. The largest difference was observed when comparing the Northeast with Alabama, Florida, Georgia, Kentucky, Mississippi, North Carolina, South Carolina, and Tennessee (region 4; AOR, 0.70 [95% CI, 0.58-0.86]; *P* < .001) and Arkansas, Louisiana, New Mexico, Oklahoma, and Texas (region 6; AOR, 0.56 [95% CI, 0.44-0.71]; *P* < .001). Compared with beneficiaries in the Northeast, only beneficiaries in Alaska, Idaho, Oregon, and Washington (region 10) had a higher adjusted odds of receiving gender-affirming surgery (AOR, 1.30 [95% CI, 1.05-1.61]; *P* = .02). Geographic differences remained consistent when comparing associations based on the CMS’s regional divisions and by Medicare Administrative Contractors themselves (eTables 4 and 5 in Supplement 1).^19,37^

**Table 2.** Associations of Beneficiary Characteristics With Gender-Affirming Surgery Using a Generalized Estimating Equation for Transgender and Gender Diverse Medicare Beneficiaries, January 2016 to February 2020

Category	AOR (95% CI)	*P* value
Medicare-Medicaid dual enrollment	1.14 (1.02-1.26)	.02
Age category, y		
18-30	1.58 (1.27-1.97)	<.001
31-40	1.91 (1.55-2.34)	<.001
41-50	1.75 (1.43-2.16)	<.001
51-55	1.51 (1.19-1.90)	.001
56-60	1.25 (0.99-1.58)	.06
61-65	1 [Reference]	NA
66-70	0.80 (0.65-0.98)	.03
71-75	0.62 (0.49-0.78)	<.001
>75	0.39 (0.31-0.50)	<.001
Race and ethnicity		
American Indian or Alaska Native	0.79 (0.46-1.36)	.40
Asian or Other Pacific Islander	0.80 (0.54-1.19)	.28
Black or African American	1 [Reference]	NA
Hispanic	0.98 (0.79-1.23)	.87
Non-Hispanic White	0.94 (0.82-1.09)	.44
Unknown	0.76 (0.52-1.09)	.14
Other^a^	0.31 (0.12-0.82)	.02
Original reason for Medicare entitlement		
Age	1.41 (1.16-1.71)	<.001
ESKD or ESKD and disability	0.87 (0.59-1.28)	.47
Disability	1 [Reference]	NA
CMS region		
Region 1 (Connecticut, Maine, Massachusetts, New Hampshire, Rhode Island, Vermont)	1 [Reference]	NA
Region 2 (New Jersey, New York, Puerto Rico, Virgin Islands)	0.79 (0.64-0.98)	.03
Region 3 (Delaware, District of Columbia, Maryland, Pennsylvania, Virginia, West Virginia)	0.82 (0.66-1.01)	.07
Region 4 (Alabama, Florida, Georgia, Kentucky, Mississippi, North Carolina, South Carolina, Tennessee)	0.70 (0.58-0.86)	<.001
Region 5 (Illinois, Indiana, Michigan, Minnesota, Ohio, Wisconsin)	0.88 (0.73-1.06)	.18
Region 6 (Arkansas, Louisiana, New Mexico, Oklahoma, Texas)	0.56 (0.44-0.71)	<.001
Region 7 (Iowa, Kansas, Missouri, Nebraska)	0.82 (0.64-1.06)	.14
Region 8 (Colorado, Montana, North Dakota, South Dakota, Utah, Wyoming)	0.82 (0.61-1.09)	.17
Region 9 (Arizona, California, Hawaii, Nevada, Pacific Territories)	1.04 (0.86-1.26)	.68
Region 10 (Alaska, Idaho, Oregon, Washington)	1.30 (1.05-1.61)	.02
Other or living outside the regions	1.68 (0.46-6.09)	.43
Chronic conditions, No.		
0	1 [Reference]	NA
1	1.31 (1.06-1.63)	.01
2-5	1.51 (1.30-1.75)	<.001
6-9	1.59 (1.36-1.86)	<.001
≥10	2.10 (1.79-2.46)	<.001

Abbreviations: AOR, adjusted odds ratio; CMS, Centers for Medicare & Medicaid Services; ESKD, end-stage kidney disease; NA, not applicable.

^a^ Medicare’s classification of “other” can be interpreted as a non-Hispanic race and ethnicity other than those offered.^29^

Compared with Black or African American beneficiaries, those listed as having an “other” race or ethnicity had 0.31 (95% CI, 0.12-0.82) times the adjusted odds of receiving gender-affirming surgery (*P* = .02) (Table 2).^29^ Compared with those aged 61 to 65 years, beneficiaries aged 60 years or younger had higher adjusted odds of having received gender-affirming surgery (31-40 years: AOR, 1.91 [95% CI, 1.55-2.34]). Beneficiaries aged 71 years or older had lower adjusted odds. Those dually enrolled in Medicaid had a higher adjusted odds of receiving gender-affirming surgery compared with beneficiaries only enrolled in Medicare (AOR, 1.14 [95% CI, 1.02-1.26]; *P* = .02). Beneficiaries who became eligible for Medicare due to age had a higher adjusted odds of receiving gender-affirming surgery compared with those who became eligible for Medicare due to disability (AOR, 1.41 [95% CI, 1.16-1.71]; *P* < .001). Last, TGD beneficiaries with chronic conditions had a higher adjusted odds of receiving gender-affirming surgery compared with those with no or fewer chronic conditions (≥10 conditions vs 0: AOR, 2.10 [95% CI, 1.79-2.46]; *P* < .001).

### Gender-Affirming Surgical Procedures Among the Comparison Group

The comparison group of beneficiaries (eTable 6 in Supplement 1) included 160 387 person-year observations for 101 361 non-TGD beneficiaries. There were 761 instances of a non-TGD individual having at least 1 surgical procedure in a calendar year that could be considered gender affirming. The proportion of the non-TGD cohort receiving at least 1 gender-affirming or comparable surgical procedure was between 0.5% and 0.7% (Figure 1). Both the comparison group and TGD beneficiaries received gender-affirming surgical procedures for complications related to urinary stents, prosthetic devices, and other procedures; however, the comparison group did not receive as many mammoplasties, excisions of testes, and other surgical procedures (eTable 7 in Supplement 1).

Our intention was to see if TGD beneficiaries had a differential receipt of the same surgical procedures. Therefore, in our primary analysis, we did not exclude surgical procedures indicated for cancer-related care for the non-TGD cohort, as we were examining all surgical procedures received presumably not exclusively for gender-affirming purposes. In our adjusted analysis, beneficiaries within our comparison group had similar trends in gender-affirming surgery as the TGD cohort, such as a higher adjusted odds of receiving surgery for those with dual enrollment in Medicare and Medicaid and among certain younger groups (eTable 8 in Supplement 1). Unlike the TGD sample, there were no significant differences in the frequency of surgical procedures across CMS regions for the comparison group. We also performed the same adjusted analysis, excluding surgical procedures indicated for cancer-related care, and still found no geographic differences.

## Discussion

This is the first study, to our knowledge, to compare the receipt of gender-affirming surgery by Medicare beneficiary characteristics for both TGD individuals and a non-TGD comparison group. Our inclusion of a non-TGD comparison group, as well as our use of an algorithm and our decision to exclude surgical procedures that may have been for cancer-related care, makes our methodological approach unique. We found that gender-affirming surgical procedures among TGD Medicare beneficiaries were rare, regional differences existed in the proportion of TGD beneficiaries receiving gender-affirming surgery yet not among those in the comparison group, and differing proportions of gender-affirming surgical procedures also exist by other beneficiary characteristics.

Among TGD Medicare beneficiaries, the proportion receiving at least 1 gender-affirming surgical procedure was 1.4% to 2.2% during the study period; among non-TGD individuals, the proportion receiving the same surgical procedures was 0.5% to 0.7%. Although there have been multiple studies drawn from other data sources indicating the rate of gender-affirming surgical procedures substantially increased during our study period,^13,14,15,16,18^ our findings show that the rate of receipt of gender-affirming surgical procedures was consistently low throughout the study period and decreased in 2018 and 2019. This finding is consistent with a previous study’s findings regarding the Medicare-covered population.^18^ Our findings also support those of a previous study, which found that Medicare beneficiaries have significantly lower odds of receiving gender-affirming surgery compared with those with private insurance, despite an overall increase in gender-affirming surgical procedures.^14^

Several factors could be associated with the low proportion of beneficiaries receiving gender-affirming surgical procedures in Medicare. TGD Medicare beneficiaries face barriers to receiving gender-affirming surgery because of a lack of access to surgeons, coverage denials, and high out-of-pocket costs—structural forms of stigma that may be particularly elevated for racial and ethnic minoritized populations due to racism.^20,30,36,38^ Medicare’s low reimbursement rates for gender-affirming surgery may also play a role. After adjusting for inflation, reimbursement for gender-affirming surgery has decreased by more than 10% since 2014.^39,40^ In addition, Medicare coverage of gender-affirming surgery is determined on a case-by-case basis per current CMS policy.^41^ As a result, Medicare beneficiaries may be less likely to have medically necessary services covered compared with TGD people with other forms of insurance.^38^

We also found significant geographic variation between the 10 CMS regions in the probability of gender-affirming surgery for TGD-identified Medicare beneficiaries. The results of our adjusted analysis found that, compared with those in the Northeast, TGD beneficiaries residing in many parts of the country, particularly in the South, have lower adjusted odds of receipt of gender-affirming surgery. These differences are consistent with previous studies.^13,16,36,42^ There was no significant geographic variation in the probability of receipt of the same surgeries for our non-TGD cohort. This lack of geographic variation potentially indicates unique access issues for the TGD Medicare population, which may be associated with regional contractors’ practices.

Medicaid coverage may allow for greater access to gender-affirming surgery, as dually enrolled beneficiaries had higher adjusted odds of receiving gender-affirming surgery compared with Medicare-only beneficiaries. Certain states have Medicaid programs that explicitly cover gender-affirming surgery,^18^ and Medicaid can cover Medicare’s cost-sharing.^43^ These cost-sharing protections could increase access to gender-affirming surgery, especially given the high rates of poverty in TGD populations.^44^ Furthermore, dually enrolled beneficiaries may have the added assurance of Medicaid covering gender-affirming surgery in the event of a Medicare denial. We also saw different odds by age category, original reason for Medicare entitlement, and chronic conditions, which warrants further study.

### Limitations

Our study has several limitations. First, Medicare claims data do not include self-reported gender identity. We relied on a claims-based algorithm to identify TGD individuals. This algorithm limited our TGD cohort to individuals who accessed health care and are “out” to at least 1 medical provider. Claims-based algorithms, similar to the one used by the study team, have been shown to have high sensitivity and specificity, as discussed in the Methods section.^25^ Second, our sample sizes were small relative to general Medicare population, which may have reduced power. Third, our study period was confined to the years 2016 to 2020. We recognize that observed trends could be associated with the October 2015 switch to *ICD-10* codes. Fourth, it is possible that our non-TGD cohort included people who were TGD but were not flagged by the algorithm. Fifth, we relied on Medicare race and ethnicity data, which may have misclassified and biased outcomes, especially for non-Hispanic American Indian or Alaska Native populations.^34^ Sixth, our analysis did not include those with Medicare Advantage. Medicare Advantage plans do not rely on Medicare Administrative Contractors to make coverage determinations. Future studies should compare receipt of gender-affirming surgery for those with Medicare Advantage and those with fee-for-service Medicare.

Seventh, we acknowledge that not all TGD beneficiaries wish to receive gender-affirming surgery. There are also TGD individuals who need gender-affirming surgery yet cannot access these services given the barriers put in place by Medicare, including cost-sharing, as well as other inequities associated with racism and other structural determinants of health.^20,30,36,38^ Accessing gender-affirming surgery, historically and presently, is challenging, even among individuals who are insured. In the 1960s, respectability politics (the belief that conformity to prescribed mainstream standards of appearance and behavior will protect a person who is part of a marginalized group from prejudices and systemic injustices) led to greater accessibility of gender-affirming surgery for White individuals, while others, including members of racial and ethnic minoritized populations, sex workers, justice-involved individuals, and individuals who are nonbinary, frequently faced extreme barriers to coverage.^31,32^ Research has found that inequities persist today, as those with higher incomes and health insurance more often receive gender-affirming surgery,^9,45^ and with large proportions of those receiving gender-affirming surgery being identified as White.^13^ We are mindful that those with intersecting marginalized identities, including Black TGD people, face barriers to care, even when enrolled in Medicare.^31,32^ Future qualitative research with patients and clinicians is needed to assess the mechanisms underlying our findings.

Eighth, we do not know what proportion of TGD beneficiaries received gender-affirming surgery prior to enrolling in Medicare, paid out of pocket for gender-affirming surgery, or received surgery covered by an insurer other than Medicare. We were unable to observe claims for services that took place outside of the Medicare program. However, the lack of geographic variation among non-TGD beneficiaries still indicates unique access issues for the TGD Medicare population. Nonetheless, both the non-TGD and TGD cohorts could have received gender-affirming surgical procedures outside of the Medicare program. In addition, while the general Medicare population is typically eligible based on age (ie, aged ≥65 years), TGD Medicare beneficiaries are, on average, younger, as they are more likely to qualify for Medicare due to disability.^46^ With previous studies finding that patients who receive gender-affirming surgery are frequently young adults,^13^ it is particularly concerning that gender-affirming surgery is so rare among Medicare beneficiaries.

## Conclusions

In this cross-sectional study, we found very low rates of receipt of gender-affirming surgery in the Medicare program, as well as significant variation by geography and other beneficiary characteristics (eg, race and ethnicity, age, and dual enrollment in Medicare and Medicaid). At a time when TGD communities are under legal, political, and social attack,^47,48^ it is critical to highlight the reality of the accessibility of gender-affirming care, including gender-affirming surgery. Although US citizens, regardless of sex and gender, are entitled to Medicare through their work history, and although gender-affirming surgery is a medically necessary form of care,^7,8^ TGD Medicare beneficiaries face barriers to care that their non-TGD counterparts do not. Our findings point to the need for additional studies as well as an opportunity for the CMS to ensure equitable access to needed medical care for TGD Medicare beneficiaries.

## Notes

Supplement 1. **eFigure.** Propensity Score Matching Graphs by Year, 2016-2020 **eTable 1.** Gender-Affirming Surgery Codes **eTable 2.** Cancer-Related Diagnosis Codes That Exclude Transgender and Gender Diverse Individuals From Our Analysis if Their Surgery Could Be Cancer-Related Care **eTable 3.** Rate of Gender-Affirming Surgery in CMS Regions per 1,000 Beneficiary-Years by Gender Identity, January 2016-February 2020 **eTable 4.** Associations Between Transgender Beneficiary Characteristics and Gender-Affirming Surgery, Replacing CMS Regions With Medicare Administrative Contractor Regions, January 2016-February 2020 **eTable 5.** Associations Between Transgender Beneficiary Characteristics and Gender-Affirming Surgery, Replacing CMS Region With Medicare Administrative Contractors, January 2016-February 2020 **eTable 6.** Characteristics of Beneficiaries Not Identified as Transgender or Gender Diverse Who Did and Did Not Receive Surgeries That Could Be Classified as Gender-Affirming at the Person-Year Level, January 2016–February 2020 **eTable 7.** Most Common Codes From eTable 1 Found In Surgery Claims, by Gender Modality **eTable 8.** Bivariate Associations Between Sociodemographic Factors and Surgery Using a Generalized Estimating Equation for Medicare Beneficiaries Who Are Not Identified as Transgender or Gender Diverse, January 2016-February 2020

Supplement 2.
Data Sharing Statement

## References

[1] Pellicane MJ, Ciesla JA. Associations between minority stress, depression, and suicidal ideation and attempts in transgender and gender diverse (TGD) individuals: systematic review and meta-analysis. Clin Psychol Rev. 2022;91:102113. doi:10.1016/j.cpr.2021.102113 34973649

[2] Study: Paying for transgender health care cost-effective. Johns Hopkins Bloomberg School of Public Health. December 1, 2015. Accessed July 15, 2024. https://publichealth.jhu.edu/2015/study-paying-for-transgender-health-care-cost-effective

[3] Esmonde NO, Heston AL, Morrison T, . Providing gender confirmation surgery at an academic medical center: analysis of use, insurance payer, and fiscal impact. J Am Coll Surg. 2019;229(5):479-486. doi:10.1016/j.jamcollsurg.2019.07.002 31326537

[4] Hughto JMW, Gunn HA, Rood BA, Pantalone DW. Social and medical gender affirmation experiences are inversely associated with mental health problems in a U.S. non-probability sample of transgender adults. Arch Sex Behav. 2020;49(7):2635-2647. doi:10.1007/s10508-020-01655-5 32215775 PMC7494544

[5] Almazan AN, Keuroghlian AS. Association between gender-affirming surgeries and mental health outcomes. JAMA Surg. 2021;156(7):611-618. doi:10.1001/jamasurg.2021.0952 33909023 PMC8082431

[6] Mann S, Campbell T, Nguyen DH. Access to gender-affirming care and transgender mental health: evidence from Medicaid coverage. Am J Health Econ. 2024;10(2):162-181. doi:10.1086/728080

[7] Medical association statements in support of health care for transgender people and youth. GLAAD. June 26, 2024. Accessed March 17, 2025. https://glaad.org/medical-association-statements-supporting-trans-youth-healthcare-and-against-discriminatory/

[8] Trans Health Project. Medical organization statements. Advocates for Trans Equality Inc. Accessed August 18, 2023. https://transhealthproject.org/resources/medical-organization-statements/

[9] James SE, Herman JL, Rankin S, Keisling M, Mottet L, Anafi M. *The Report of the 2015 U.S. Transgender Survey*. National Center for Transgender Equality. 2016. Accessed August 18, 2023. https://transequality.org/sites/default/files/docs/usts/USTS-Full-Report-Dec17.pdf

[10] Kirzinger A, Kearney A, Montero A, Sparks G, Dawson L, Brodie M. KFF/*The Washington Post* Trans Survey. KFF. March 24, 2023. Accessed May 13, 2024. https://www.kff.org/report-section/kff-the-washington-post-trans-survey-trans-in-america/

[11] Canner JK, Harfouch O, Kodadek LM, . Temporal trends in gender-affirming surgery among transgender patients in the United States. JAMA Surg. 2018;153(7):609-616. doi:10.1001/jamasurg.2017.6231 29490365 PMC5875299

[12] Lane M, Ives GC, Sluiter EC, . Trends in gender-affirming surgery in insured patients in the United States. Plast Reconstr Surg Glob Open. 2018;6(4):e1738. doi:10.1097/GOX.0000000000001738 29876180 PMC5977951

[13] Wright JD, Chen L, Suzuki Y, Matsuo K, Hershman DL. National estimates of gender-affirming surgery in the US. JAMA Netw Open. 2023;6(8):e2330348. doi:10.1001/jamanetworkopen.2023.30348 37610753 PMC10448302

[14] Chu J, Nagpal M, Dobberfuhl AD. Utilization and cost of gender-affirming surgery in the United States from 2012-2019. Ann Surg. 2025;281(5):814-822. doi:10.1097/SLA.0000000000006296 38618736 PMC11473710

[15] Fazal M, Oles N, Beckham SW, . Sociodemographics of patient populations undergoing gender-affirming surgery: a systematic review of all cohort studies. Transgend Health. 2023;8(3):213-219. doi:10.1089/trgh.2021.0111 37342473 PMC10278024

[16] Das RK, Galdyn I, McCaffrey RL, Drolet BC, Al Kassis S. Geographic differences in patient demographics and performance of gender-affirming surgery from 2016 to 2019. Aesthet Surg J. 2024;44(3):NP209-NP217. doi:10.1093/asj/sjad353 37995314

[17] Baker K, Restar A. Utilization and costs of gender-affirming care in a commercially insured transgender population. J Law Med Ethics. 2022;50(3):456-470. doi:10.1017/jme.2022.87 36398652 PMC9679590

[18] Faletsky A, Jonczyk MM, Guo L. Legislations mandating insurance coverage are highly effective in delivering surgical care of transgender patients. Plast Reconstr Surg Glob Open. 2022;10(8):e4496. doi:10.1097/GOX.0000000000004496 36061492 PMC9433082

[19] Who are the MACs. Centers for Medicare & Medicaid Services. Accessed August 22, 2024. https://www.cms.gov/medicare/coding-billing/medicare-administrative-contractors-macs/who-are-macs

[20] Bakko M, Kattari SK. Differential access to transgender inclusive insurance and healthcare in the United States: challenges to health across the life course. J Aging Soc Policy. 2021;33(1):67-81. doi:10.1080/08959420.2019.1632681 31230581

[21] MEDPAR limited data set (LDS)—hospital (national). Centers for Medicare & Medicaid Services. Accessed October 18, 2023. https://www.cms.gov/data-research/files-for-order/limited-data-set-lds-files/medpar-limited-data-set-lds-hospital-national

[22] Outpatient (fee-for-service). Research Data Assistance Center. Accessed October 18, 2023. https://resdac.org/cms-data/files/op-ffs

[23] Master Beneficiary Summary File (MBSF) LDS. Centers for Medicare & Medicaid Services. Accessed October 18, 2023. https://www.cms.gov/data-research/files-for-order/limited-data-set-lds-files/master-beneficiary-summary-file-mbsf-lds

[24] Hughto JMW, Hughes L, Yee K, . Improving data-driven methods to identify and categorize transgender individuals by gender in insurance claims data. LGBT Health. 2022;9(4):254-263. doi:10.1089/lgbt.2021.0433 35290746 PMC9150133

[25] Streed CG, King D, Grasso C, et al. Validation of an administrative algorithm for transgender and gender diverse persons against self-report data in electronic health records. J Am Med Inform Assoc JAMIA. 2023;30(6):1047-1055. doi:10.1093/jamia/ocad039 PMC10198536 36921287

[26] CMS Regional Offices. Centers for Medicare & Medicaid Services. Accessed October 18, 2023. https://www.cms.gov/about-cms/where-we-are/regional-offices

[27] Billing and coding: gender reassignment services for gender dysphoria (A53793). Centers for Medicare & Medicaid Services. Accessed August 22, 2024. https://www.cms.gov/medicare-coverage-database/view/article.aspx?articleid=53793

[28] Squires LR, Bilash T, Kamen CS, Garland SN. Psychosocial needs and experiences of transgender and gender diverse people with cancer: a scoping review and recommendations for improved research and care. LGBT Health. 2022;9(1):8-17. doi:10.1089/lgbt.2021.0072 34495755 PMC9206485

[29] Huang AW, Meyers DJ. Assessing the validity of race and ethnicity coding in administrative Medicare data for reporting outcomes among Medicare Advantage beneficiaries from 2015 to 2017. Health Serv Res. 2023;58(5):1045-1055. doi:10.1111/1475-6773.14197 37356821 PMC10480088

[30] Lett E, Asabor E, Beltrán S, Cannon AM, Arah OA. Conceptualizing, contextualizing, and operationalizing race in quantitative health sciences research. Ann Fam Med. 2022;20(2):157-163. doi:10.1370/afm.2792 35045967 PMC8959750

[31] Joyner C. Kadji Amin presents “The Respectability Politics of Gender Identity.” Department of Gender, Sexuality & Feminist Studies; Duke Trinity College of Arts and Sciences. October 30, 2023. Accessed July 16, 2024. https://gendersexualityfeminist.duke.edu/news/kadji-amin-presents-respectability-politics-gender-identity

[32] Stryker S. *Transgender History: The Roots of Today’s Revolution*. 2nd ed. Seal Press; 2017. Accessed July 16, 2024. https://www.hachettebookgroup.com/titles/susan-stryker/transgender-history-second-edition/9781580056908/?lens=seal-press

[33] MBSF 30 CCW chronic conditions. Research Data Assistance Center. Accessed February 12, 2025. https://resdac.org/cms-data/files/mbsf-30-cc

[34] MBSF other chronic or potentially disabling conditions. Research Data Assistance Center. Accessed February 12, 2025. https://resdac.org/cms-data/files/mbsf-other-conditions

[35] Hanley JA, Negassa A, Edwardes MD, Forrester JE. Statistical analysis of correlated data using generalized estimating equations: an orientation. Am J Epidemiol. 2003;157(4):364-375. doi:10.1093/aje/kwf215 12578807

[36] Terris-Feldman A, Chen A, Poudrier G, Garcia M. How accessible is genital gender-affirming surgery for transgender patients with commercial and public health insurance in the United States? results of a patient-modeled search for services and a survey of providers. Sex Med. 2020;8(4):664-672. doi:10.1016/j.esxm.2020.08.005 33023854 PMC7691873

[37] MAC Directory. National Government Services. Accessed August 22, 2024. https://www.ngsmedicare.com/mac-directory

[38] Bakko M, Kattari SK. Transgender-related insurance denials as barriers to transgender healthcare: differences in experience by insurance type. J Gen Intern Med. 2020;35(6):1693-1700. doi:10.1007/s11606-020-05724-2 32128693 PMC7280420

[39] Cordero JJ, Alaniz L, Kalavacherla S, Tholpady SS, Chu MW. Trends of Medicare reimbursement rates for gender-affirming surgery procedures. Ann Plast Surg. 2024;92(5S suppl 3):S366-S370. doi:10.1097/SAP.0000000000003799 38689421

[40] Siotos C, Underhill JM, Sykes J, . Trends of Medicare reimbursement rates for gender affirmation procedures. J Sex Med. 2024;21(2):181-191. doi:10.1093/jsxmed/qdad160 38055925

[41] Ngaage LM, Xue S, Borrelli MR, . Gender-affirming health insurance reform in the United States. Ann Plast Surg. 2021;87(2):119-122. doi:10.1097/SAP.0000000000002674 33470627

[42] Dagi AF, Boskey ER, Nuzzi LC, . Legislation, market size, and access to gender-affirming genital surgery in the United States. Plast Reconstr Surg Glob Open. 2021;9(2):e3422. doi:10.1097/GOX.0000000000003422 33680670 PMC7929723

[43] Roberts ET, Desai SM. Does Medicaid coverage of Medicare cost sharing affect physician care for dual-eligible Medicare beneficiaries? Health Serv Res. 2021;56(3):528-539. doi:10.1111/1475-6773.13650 33778957 PMC8143678

[44] Wilson BDM, Bouton LJA, Badgett MVL, Macklin ML. LGBT poverty in the United States: trends at the onset of COVID-19. UCLA School of Law Williams Institute. February 2023. Accessed April 7, 2024. https://williamsinstitute.law.ucla.edu/publications/lgbt-poverty-us/

[45] Kim EJ, Stearns SA, Bustos VP, Dowlatshahi AS, Lee BT, Cauley R. Impact of financial well-being on gender affirmation surgery access and hospital course. J Plast Reconstr Aesthet Surg. 2023;85:174-181. doi:10.1016/j.bjps.2023.06.059 37499558

[46] Dragon CN, Guerino P, Ewald E, Laffan AM. Transgender Medicare beneficiaries and chronic conditions: exploring fee-for-service claims data. LGBT Health. 2017;4(6):404-411. doi:10.1089/lgbt.2016.0208 29125908 PMC5731542

[47] Owermohle S, Wilkerson J, Zhang RC, Lawrence L. Trump’s initial orders reverse Biden on health care costs, protections from discrimination. STAT. January 20, 2025. Accessed January 22, 2025. https://www.statnews.com/2025/01/20/trump-executive-orders-health-care-drug-pricing-aca-covid-gender-discrimination/

[48] Reyes EA. Trump orders federal agencies to recognize only two sexes that are “not changeable.” *Los Angeles Times*. January 21, 2025. Accessed January 22, 2025. https://www.latimes.com/california/story/2025-01-21/trump-issues-executive-order-to-recognize-only-two-genders

