# Supplementary material for: Gender-Affirming Surgery for Transgender and Gender Diverse Medicare Beneficiaries
Source: JAMA Netw Open. 2025 May 1;8(5):e258072. doi: 10.1001/jamanetworkopen.2025.8072 (PMC12046427; doi:10.1001/jamanetworkopen.2025.8072)
Supplement: Supplement 1. — eFigure. Propensity Score Matching Graphs by Year, 2016-2020 eTable 1. Gender-Affirming Surgery Codes eTable 2. Cancer-Related Diagnosis Codes That Exclude Transgender and Gender Diverse Individuals From Our Analysis if Their Surgery Could Be Cancer-Related Care eTable 3. Rate of Gender-Affirming Surgery in CMS Regions per 1,000 Beneficiary-Years by Gender Identity, January 2016-February 2020 eTable 4. Associations Between Transgender Beneficiary Characteristics and Gender-Affirming Surgery, Replacing CMS Regions With Medicare Administrative Contractor Regions, January 2016-February 2020 eTable 5. Associations Between Transgender Beneficiary Characteristics and Gender-Affirming Surgery, Replacing CMS Region With Medicare Administrative Contractors, January 2016-February 2020 eTable 6. Characteristics of Beneficiaries Not Identified as Transgender or Gender Diverse Who Did and Did Not Receive Surgeries That Could Be Classified as Gender-Affirming at the Person-Year Level, January 2016–February 2020 eTable 7. Most Common Codes From eTable 1 Found In Surgery Claims, by Gender Modality eTable 8. Bivariate Associations Between Sociodemographic Factors and Surgery Using a Generalized Estimating Equation for Medicare Beneficiaries Who Are Not Identified as Transgender or Gender Diverse, January 2016-February 2020 [file jamanetwopen-e258072-s001.pdf]

Supplementary Online Content

Balkan E, Babbs G, Meyers DJ, et al. Gender-affirming surgery for transgender and gender diverse Medicare beneficiaries. *JAMA Netw Open.* 2025;8(5):e258072. doi:10.1001/jamanetworkopen.2025.8072

eFigure. Propensity Score Matching Graphs by Year, 2016-2020
eTable 1. Gender Affirming Surgery Codes
eTable 2. Cancer-Related Diagnosis Codes That Exclude Transgender and Gender Diverse Individuals from Our Analysis if Their Surgery Could Be Cancer-Related Care
eTable 3. Rate of Gender-Affirming Surgery in CMS Regions per 1,000 Beneficiary-Years by Gender Identity, January 2016-February 2020
eTable 4. Associations between Transgender Beneficiary Characteristics and Gender-Affirming Surgery, Replacing CMS Regions with Medicare Administrative Contractor Regions, January 2016-February 2020
eTable 5. Associations between Transgender Beneficiary Characteristics and Gender-Affirming Surgery, Replacing CMS Region with Medicare Administrative Contractors, January 2016-February 2020
eTable 6. Characteristics of Beneficiaries Not Identified as Transgender or Gender Diverse Who Did and Did Not Receive Surgeries That Could Be Classified as Gender-Affirming at the Person-Year Level, January 2016–February 2020
eTable 7. Most Common Codes From eTable 1 Found in Surgery Claims, by Gender Modality
eTable 8. Bivariate Associations between Sociodemographic Factors and Surgery Using a Generalized Estimating Equation for Medicare Beneficiaries Who Are Not Identified as Transgender or Gender Diverse, January 2016-February 2020

This supplementary material has been provided by the authors to give readers additional information about their work.

© 2025 Balkan E et al. *JAMA Network Open.*

eFigure. Propensity score matching graphs by year, 2016-2020

eFigure 1a. 2016

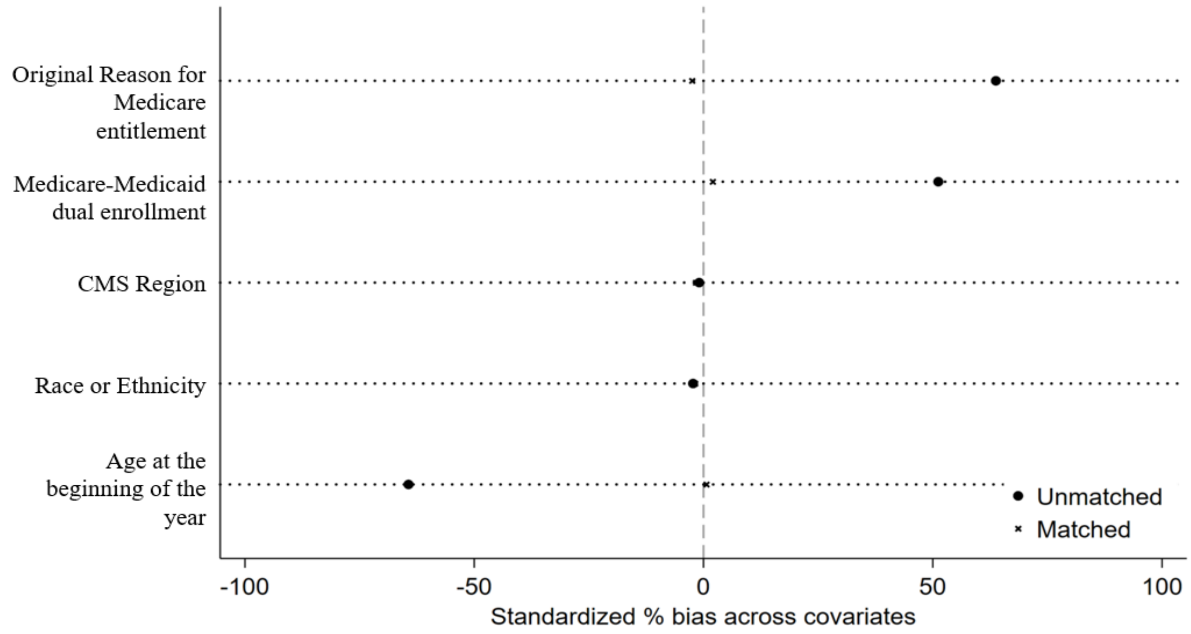

eFigure 1b. 2017

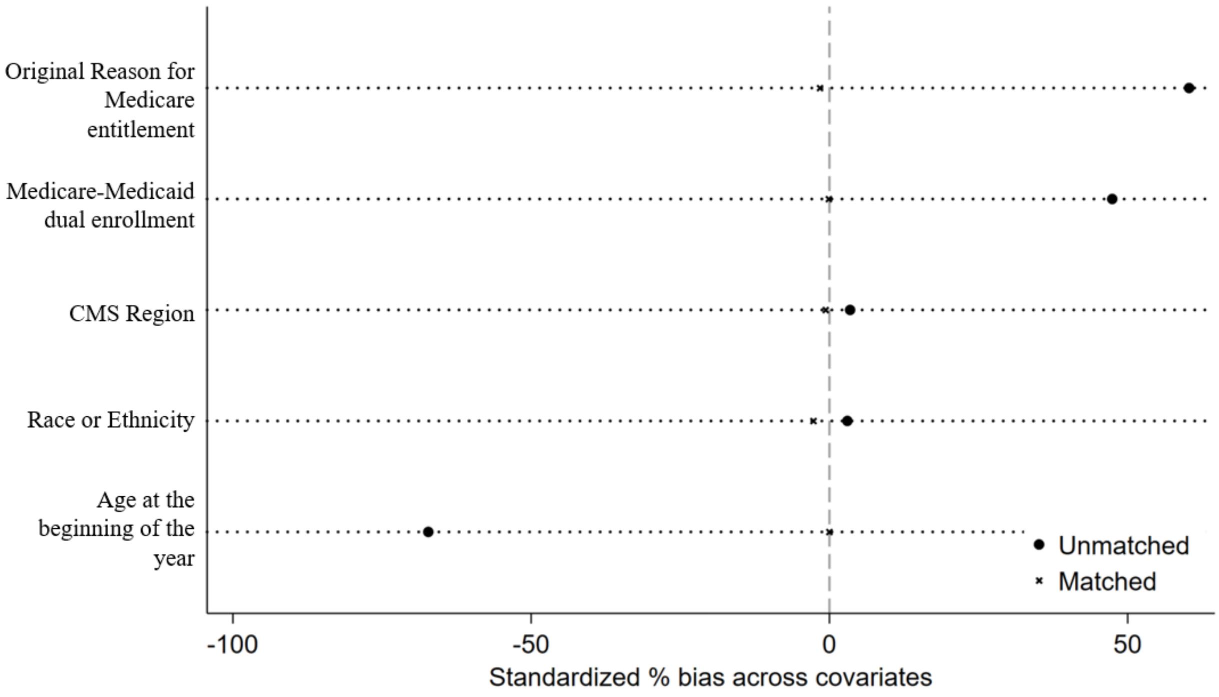

© 2025 Balkan E et al. *JAMA Network Open.*

eFigure 1c. 2018

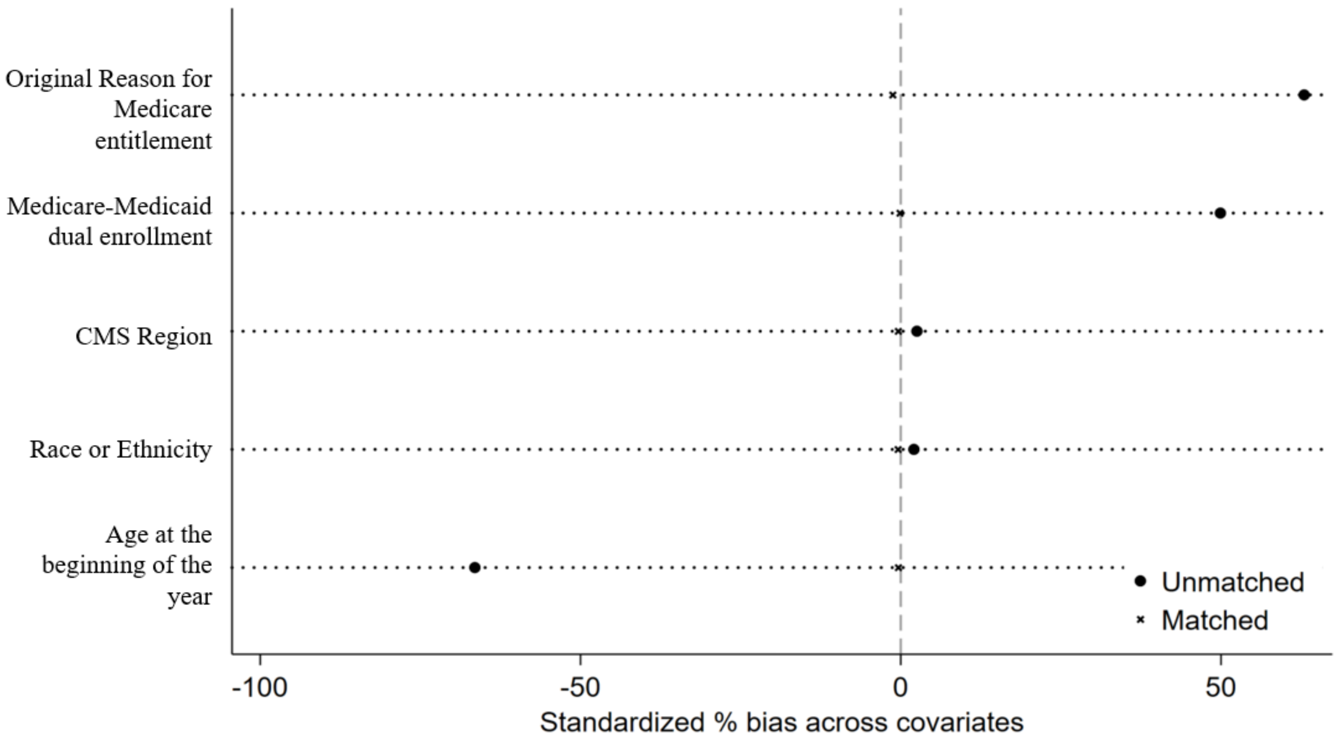

eFigure 1d. 2019

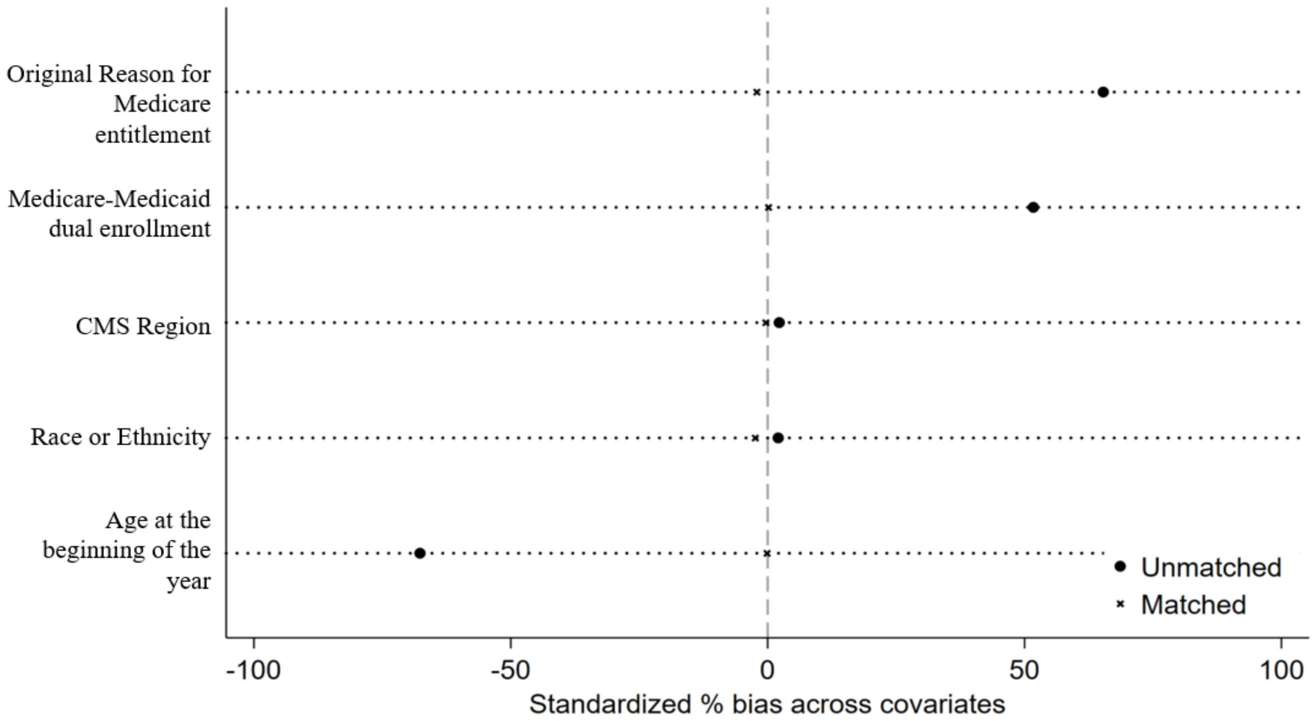

© 2025 Balkan E et al. *JAMA Network Open.*

eFigure 1e. 2020

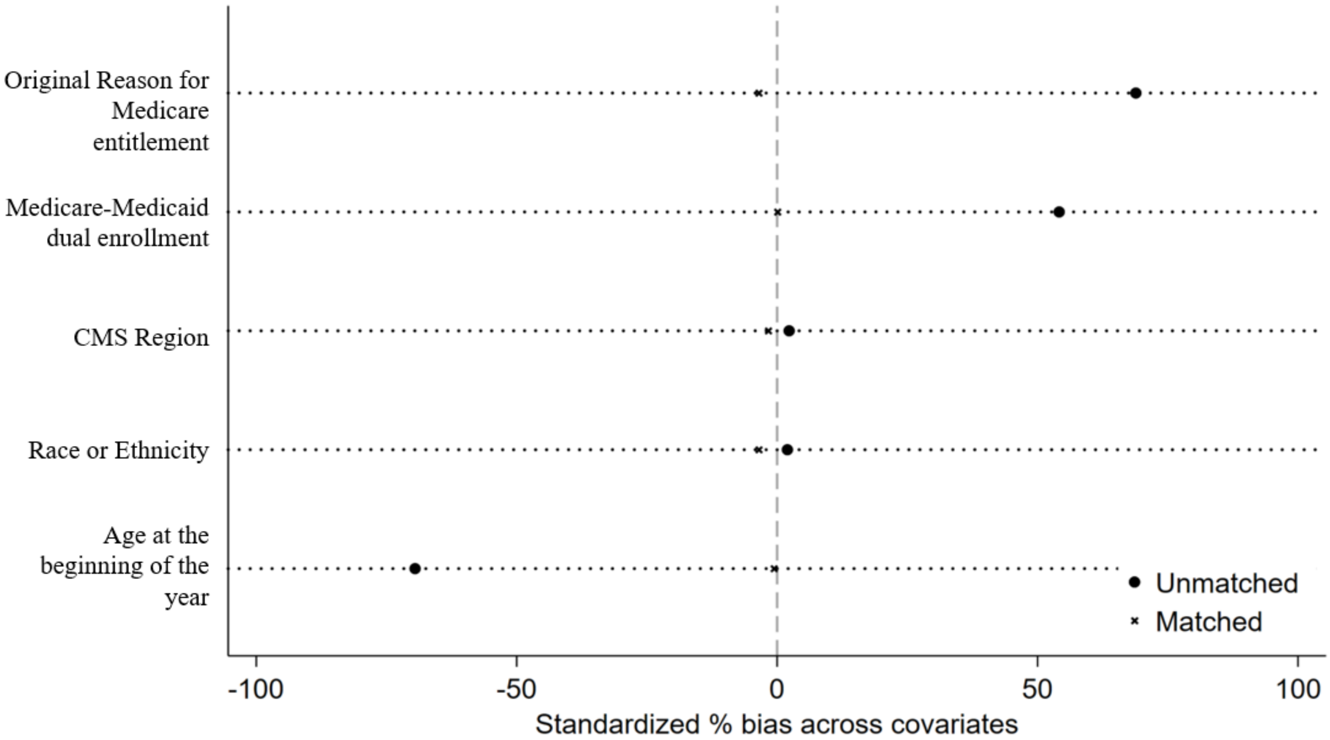

© 2025 Balkan E et al. *JAMA Network Open.*

eTable 1. Gender Affirming Surgery Codes

Code type	Code	Code description
ICD-10	0H0T07Z	Alteration of right breast with autologous tissue substitute, open approach
ICD-10	0H0T0JZ	Alteration of right breast with synthetic substitute, open approach
ICD-10	0H0T0KZ	Alteration of right breast with nonautologous tissue substitute, open approach
ICD-10	0H0T0ZZ	Alteration of right breast, open approach
ICD-10	0H0T37Z	Alteration of right breast with autologous tissue substitute, percutaneous approach
ICD-10	0H0T3JZ	Alteration of right breast with synthetic substitute, percutaneous approach
ICD-10	0H0T3KZ	Alteration of right breast with nonautologous tissue substitute, percutaneous approach
ICD-10	0H0T3ZZ	Alteration of right breast, percutaneous approach
ICD-10	0H0T3ZZ	Alteration of right breast, percutaneous approach
ICD-10	0H0TX7Z	Alteration of right breast with autologous tissue substitute, external approach
ICD-10	0H0TXJZ	Alteration of right breast with synthetic substitute, external approach
ICD-10	0H0TXKZ	Alteration of right breast with nonautologous tissue substitute, external approach
ICD-10	0H0TXZZ	Alteration of right breast, external approach
ICD-10	0H0U07Z	Alteration of left breast with autologous tissue substitute, open approach
ICD-10	0H0U0JZ	Alteration of left breast with synthetic substitute, open approach
ICD-10	0H0U0KZ	Alteration of left breast with nonautologous tissue substitute, open approach
ICD-10	0H0U0ZZ	Alteration of left breast, open approach
ICD-10	0H0U37Z	Alteration of left breast with autologous tissue substitute, percutaneous approach
ICD-10	0H0U3JZ	Alteration of left breast with synthetic substitute, percutaneous approach
ICD-10	0H0U3KZ	Alteration of left breast with nonautologous tissue substitute, percutaneous approach
ICD-10	0H0U3ZZ	Alteration of left breast, percutaneous approach
ICD-10	0H0UX7Z	Alteration of left breast with autologous tissue substitute, external approach
ICD-10	0H0UXJZ	Alteration of left breast with synthetic substitute, external approach
ICD-10	0H0UXKZ	Alteration of left breast with nonautologous tissue substitute, external approach
ICD-10	0H0UXZZ	Alteration of left breast, external approach
ICD-10	0H0V07Z	Alteration of bilateral breast with autologous tissue substitute, open approach
ICD-10	0H0V0JZ	Alteration of bilateral breast with synthetic substitute, open approach
ICD-10	0H0V0KZ	Alteration of bilateral breast with nonautologous tissue substitute, open approach
ICD-10	0H0V0ZZ	Alteration of bilateral breast, open approach
ICD-10	0H0V37Z	Alteration of bilateral breast with autologous tissue substitute, percutaneous approach
ICD-10	0H0V3JZ	Alteration of bilateral breast with synthetic substitute, percutaneous approach
ICD-10	0H0V3KZ	Alteration of bilateral breast with nonautologous tissue substitute, percutaneous approach
ICD-10	0H0V3ZZ	Alteration of bilateral breast, percutaneous approach
ICD-10	0H0VX7Z	Alteration of bilateral breast with autologous tissue substitute, external approach
ICD-10	0H0VXJZ	Alteration of bilateral breast with synthetic substitute, external approach
ICD-10	0H0VXKZ	Alteration of bilateral breast with nonautologous tissue substitute, external approach
ICD-10	0H5T0ZZ	Destruction of right breast, open approach
ICD-10	0H5T3ZZ	Destruction of right breast, percutaneous approach
ICD-10	0H5T7ZZ	Destruction of right breast, via natural or artificial opening
ICD-10	0H5T8ZZ	Destruction of right breast, via natural or artificial opening endoscopic
ICD-10	0H5TXZZ	Destruction of right breast, external approach
ICD-10	0H5U0ZZ	Destruction of left breast, open approach
ICD-10	0H5U3ZZ	Destruction of left breast, percutaneous approach
ICD-10	0H5U7ZZ	Destruction of left breast, via natural or artificial opening
ICD-10	0H5U8ZZ	Destruction of left breast, via natural or artificial opening endoscopic
ICD-10	0H5UXZZ	Destruction of left breast, external approach
ICD-10	0H5V0ZZ	Destruction of bilateral breast, open approach
ICD-10	0H5V3ZZ	Destruction of bilateral breast, percutaneous approach
ICD-10	0H5V7ZZ	Destruction of bilateral breast, via natural or artificial opening
ICD-10	0H5V8ZZ	Destruction of bilateral breast, via natural or artificial opening endoscopic
ICD-10	0H5VXZZ	Destruction of bilateral breast, external approach
ICD-10	0HBV0ZZ	Excision of bilateral breast, open approach

© 2025 Balkan E et al. *JAMA Network Open.*

ICD-10	0HBV3ZZ	Excision of bilateral breast, percutaneous approach
ICD-10	0HBV7ZZ	Excision of bilateral breast, via natural or artificial opening
ICD-10	0HBV8ZZ	Excision of bilateral breast, via natural or artificial opening endoscopic
ICD-10	0HBVXZZ	Excision of bilateral breast, external approach
ICD-10	0HBW0ZZ	Excision of right nipple, open approach
ICD-10	0HBX0ZZ	Excision of left nipple, open approach
ICD-10	0HHT0NZ	Insertion of tissue expander into right breast, open approach
ICD-10	0HHT3NZ	Insertion of tissue expander into right breast, percutaneous approach
ICD-10	0HHT7NZ	Insertion of tissue expander into right breast, via natural or artificial opening
ICD-10	0HHT8NZ	Insertion of tissue expander into right breast, via natural or artificial opening endoscopic
ICD-10	0HHU0NZ	Insertion of tissue expander into left breast, open approach
ICD-10	0HHU3NZ	Insertion of tissue expander into left breast, percutaneous approach
ICD-10	0HHU7NZ	Insertion of tissue expander into left breast, via natural or artificial opening
ICD-10	0HHU8NZ	Insertion of tissue expander into left breast, via natural or artificial opening endoscopic
ICD-10	0HHV0NZ	Insertion of tissue expander into bilateral breast, open approach
ICD-10	0HHV3NZ	Insertion of tissue expander into bilateral breast, percutaneous approach
ICD-10	0HHV7NZ	Insertion of tissue expander into bilateral breast, via natural or artificial opening
ICD-10	0HHV8NZ	Insertion of tissue expander into bilateral breast, via natural or artificial opening endoscopic
ICD-10	0HHW0NZ	Insertion of tissue expander into right nipple, open approach
ICD-10	0HHW3NZ	Insertion of tissue expander into right nipple, percutaneous approach
ICD-10	0HHW7NZ	Insertion of tissue expander into right nipple, via natural or artificial opening
ICD-10	0HHW8NZ	Insertion of tissue expander into right nipple, via natural or artificial opening endoscopic
ICD-10	0HHX0NZ	Insertion of tissue expander into left nipple, open approach
ICD-10	0HHX3NZ	Insertion of tissue expander into left nipple, percutaneous approach
ICD-10	0HHX7NZ	Insertion of tissue expander into left nipple, via natural or artificial opening
ICD-10	0HHX8NZ	Insertion of tissue expander into left nipple, via natural or artificial opening endoscopic
ICD-10	0HMTXZZ	Reattachment of right breast, external approach
ICD-10	0HMUXZZ	Reattachment of left breast, external approach
ICD-10	0HMVXZZ	Reattachment of bilateral breast, external approach
ICD-10	0HMWXZZ	Reattachment of right nipple, external approach
ICD-10	0HMXXZZ	Reattachment of left nipple, external approach
ICD-10	0HNT0ZZ	Release right breast, open approach
ICD-10	0HNT3ZZ	Release right breast, percutaneous approach
ICD-10	0HNT7ZZ	Release right breast, via natural or artificial opening
ICD-10	0HNT8ZZ	Release right breast, via natural or artificial opening endoscopic
ICD-10	0HNTXZZ	Release right breast, external approach
ICD-10	0HNU0ZZ	Release left breast, open approach
ICD-10	0HNU3ZZ	Release left breast, percutaneous approach
ICD-10	0HNU7ZZ	Release left breast, via natural or artificial opening
ICD-10	0HNU8ZZ	Release left breast, via natural or artificial opening endoscopic
ICD-10	0HNUXZZ	Release left breast, external approach
ICD-10	0HNV0ZZ	Release bilateral breast, open approach
ICD-10	0HNV3ZZ	Release bilateral breast, percutaneous approach
ICD-10	0HNV7ZZ	Release bilateral breast, via natural or artificial opening
ICD-10	0HNV8ZZ	Release bilateral breast, via natural or artificial opening endoscopic
ICD-10	0HNVXZZ	Release bilateral breast, external approach
ICD-10	0HNW0ZZ	Release right nipple, open approach
ICD-10	0HNW3ZZ	Release right nipple, percutaneous approach
ICD-10	0HNW7ZZ	Release right nipple, via natural or artificial opening
ICD-10	0HNW8ZZ	Release right nipple, via natural or artificial opening endoscopic
ICD-10	0HNWXZZ	Release right nipple, external approach
ICD-10	0HNX0ZZ	Release left nipple, open approach
ICD-10	0HNX3ZZ	Release left nipple, percutaneous approach

ICD-10	0HNX7ZZ	Release left nipple, via natural or artificial opening
ICD-10	0HNX8ZZ	Release left nipple, via natural or artificial opening endoscopic
ICD-10	0HNXXZZ	Release left nipple, external approach
ICD-10	0HPT0NZ	Removal of tissue expander from right breast, open approach
ICD-10	0HPT3NZ	Removal of tissue expander from right breast, percutaneous approach
ICD-10	0HPU0NZ	Removal of tissue expander from left breast, open approach
ICD-10	0HPU3NZ	Removal of tissue expander from left breast, percutaneous approach
ICD-10	0HQT0ZZ	Repair right breast, open approach
ICD-10	0HQT3ZZ	Repair right breast, percutaneous approach
ICD-10	0HQT7ZZ	Repair right breast, via natural or artificial opening
ICD-10	0HQT8ZZ	Repair right breast, via natural or artificial opening endoscopic
ICD-10	0HQTXZZ	Repair right breast, external approach
ICD-10	0HQU0ZZ	Repair left breast, open approach
ICD-10	0HQU3ZZ	Repair left breast, percutaneous approach
ICD-10	0HQU7ZZ	Repair left breast, via natural or artificial opening
ICD-10	0HQU8ZZ	Repair left breast, via natural or artificial opening endoscopic
ICD-10	0HQUXZZ	Repair left breast, external approach
ICD-10	0HQV	Mastopexy or nipple reconstruction
ICD-10	0HQV0ZZ	Repair bilateral breast, open approach
ICD-10	0HQV3ZZ	Repair bilateral breast, percutaneous approach
ICD-10	0HQV7ZZ	Repair bilateral breast, via natural or artificial opening
ICD-10	0HQV8ZZ	Repair bilateral breast, via natural or artificial opening endoscopic
ICD-10	0HQVXZZ	Repair bilateral breast, external approach
ICD-10	0HQW0ZZ	Repair right nipple, open approach
ICD-10	0HQW3ZZ	Repair right nipple, percutaneous approach
ICD-10	0HQW7ZZ	Repair right nipple, via natural or artificial opening
ICD-10	0HQW8ZZ	Repair right nipple, via natural or artificial opening endoscopic
ICD-10	0HQWXZZ	Repair right nipple, external approach
ICD-10	0HQX0ZZ	Repair left nipple, open approach
ICD-10	0HQX3ZZ	Repair left nipple, percutaneous approach
ICD-10	0HQX7ZZ	Repair left nipple, via natural or artificial opening
ICD-10	0HQX8ZZ	Repair left nipple, via natural or artificial opening endoscopic
ICD-10	0HQXXZZ	Repair left nipple, external approach
ICD-10	0HQY0ZZ	Repair supernumerary breast, open approach
ICD-10	0HQY3ZZ	Repair supernumerary breast, percutaneous approach
ICD-10	0HQY7ZZ	Repair supernumerary breast, via natural or artificial opening
ICD-10	0HQY8ZZ	Repair supernumerary breast, via natural or artificial opening endoscopic
ICD-10	0HQYXZZ	Repair supernumerary breast, external approach
ICD-10	0HRT07Z	Replacement of right breast with autologous tissue substitute, open approach
ICD-10	0HRT0JZ	Replacement of right breast with synthetic substitute, open approach
ICD-10	0HRT0KZ	Replacement of right breast with nonautologous tissue substitute, open approach
ICD-10	0HRT37Z	Replacement of right breast with autologous tissue substitute, percutaneous approach
ICD-10	0HRT3JZ	Replacement of right breast with synthetic substitute, percutaneous approach
ICD-10	0HRT3KZ	Replacement of right breast with nonautologous tissue substitute, percutaneous approach
ICD-10	0HRTX7Z	Replacement of right breast with autologous tissue substitute, external approach
ICD-10	0HRTXJZ	Replacement of right breast with synthetic substitute, external approach
ICD-10	0HRTXKZ	Replacement of right breast with nonautologous tissue substitute, external approach
ICD-10	0HRU07Z	Replacement of left breast with autologous tissue substitute, open approach
ICD-10	0HRU0JZ	Replacement of left breast with synthetic substitute, open approach
ICD-10	0HRU0KZ	Replacement of left breast with nonautologous tissue substitute, open approach
ICD-10	0HRU37Z	Replacement of left breast with autologous tissue substitute, percutaneous approach
ICD-10	0HRU3JZ	Replacement of left breast with synthetic substitute, percutaneous approach
ICD-10	0HRU3KZ	Replacement of left breast with nonautologous tissue substitute, percutaneous approach
ICD-10	0HRUX7Z	Replacement of left breast with autologous tissue substitute, external approach
ICD-10	0HRUXJZ	Replacement of left breast with synthetic substitute, external approach

© 2025 Balkan E et al. *JAMA Network Open.*

ICD-10	0HRUXKZ	Replacement of left breast with nonautologous tissue substitute, external approach
ICD-10	0HRV07Z	Replacement of bilateral breast with autologous tissue substitute, open approach
ICD-10	0HRV07Z	Replacement of bilateral breast with autologous tissue substitute, open approach
ICD-10	0HRV0JZ	Replacement of bilateral breast with synthetic substitute, open approach
ICD-10	0HRV0KZ	Replacement of bilateral breast with nonautologous tissue substitute, open approach
ICD-10	0HRV37Z	Replacement of bilateral breast with autologous tissue substitute, percutaneous approach
ICD-10	0HRV3JZ	Replacement of bilateral breast with synthetic substitute, percutaneous approach
ICD-10	0HRV3KZ	Replacement of bilateral breast with nonautologous tissue substitute, percutaneous approach
ICD-10	0HRVX7Z	Replacement of bilateral breast with autologous tissue substitute, external approach
ICD-10	0HRVXJZ	Replacement of bilateral breast with synthetic substitute, external approach
ICD-10	0HRVXKZ	Replacement of bilateral breast with nonautologous tissue substitute, external approach
ICD-10	0HRW07Z	Replacement of right nipple with autologous tissue substitute, open approach
ICD-10	0HRW0JZ	Replacement of right nipple with synthetic substitute, open approach
ICD-10	0HRW0KZ	Replacement of right nipple with nonautologous tissue substitute, open approach
ICD-10	0HRW37Z	Replacement of right nipple with autologous tissue substitute, percutaneous approach
ICD-10	0HRW3JZ	Replacement of right nipple with synthetic substitute, percutaneous approach
ICD-10	0HRW3KZ	Replacement of right nipple with nonautologous tissue substitute, percutaneous approach
ICD-10	0HRWX7Z	Replacement of right nipple with autologous tissue substitute, external approach
ICD-10	0HRWXJZ	Replacement of right nipple with synthetic substitute, external approach
ICD-10	0HRWXKZ	Replacement of right nipple with nonautologous tissue substitute, external approach
ICD-10	0HRX07Z	Replacement of left nipple with autologous tissue substitute, open approach
ICD-10	0HRX0JZ	Replacement of left nipple with synthetic substitute, open approach
ICD-10	0HRX0KZ	Replacement of left nipple with nonautologous tissue substitute, open approach
ICD-10	0HRX37Z	Replacement of left nipple with autologous tissue substitute, percutaneous approach
ICD-10	0HRX3JZ	Replacement of left nipple with synthetic substitute, percutaneous approach
ICD-10	0HRX3KZ	Replacement of left nipple with nonautologous tissue substitute, percutaneous approach
ICD-10	0HRXX7Z	Replacement of left nipple with autologous tissue substitute, external approach
ICD-10	0HRXXJZ	Replacement of left nipple with synthetic substitute, external approach
ICD-10	0HRXXKZ	Replacement of left nipple with nonautologous tissue substitute, external approach
ICD-10	0HSV0	Mastopexy or nipple reconstruction
ICD-10	0HSV0ZZ	Reposition bilateral breast, open approach
ICD-10	0HSWXZZ	Reposition right nipple, external approach
ICD-10	0HSXXZZ	Reposition left nipple, external approach
ICD-10	0HTT0ZZ	Resection of right breast, open approach
ICD-10	0HTU0ZZ	Resection of left breast, open approach
ICD-10	0HTV0ZZ	Resection of bilateral breast, open approach
ICD-10	0HUT07Z	Supplement right breast with autologous tissue substitute, open approach
ICD-10	0HUT0JZ	Supplement right breast with synthetic substitute, open approach
ICD-10	0HUT0KZ	Supplement right breast with nonautologous tissue substitute, open approach
ICD-10	0HUT37Z	Supplement right breast with autologous tissue substitute, percutaneous approach
ICD-10	0HUT3JZ	Supplement right breast with synthetic substitute, percutaneous approach
ICD-10	0HUT3KZ	Supplement right breast with nonautologous tissue substitute, percutaneous approach
ICD-10	0HUT77Z	Supplement right breast with autologous tissue substitute, via natural or artificial opening
ICD-10	0HUT7JZ	Supplement right breast with synthetic substitute, via natural or artificial opening
ICD-10	0HUT7KZ	Supplement right breast with nonautologous tissue substitute, via natural or artificial opening
ICD-10	0HUT87Z	Supplement right breast with autologous tissue substitute, via natural or artificial opening endoscopic
ICD-10	0HUT8JZ	Supplement right breast with synthetic substitute, via natural or artificial opening endoscopic

ICD-10	0HUT8KZ	Supplement right breast with nonautologous tissue substitute, via natural or artificial opening endoscopic
ICD-10	0HUTX7Z	Supplement right breast with autologous tissue substitute, external approach
ICD-10	0HUTXJZ	Supplement right breast with synthetic substitute, external approach
ICD-10	0HUTXKZ	Supplement right breast with nonautologous tissue substitute, external approach
ICD-10	0HUU07Z	Supplement left breast with autologous tissue substitute, open approach
ICD-10	0HUU0JZ	Supplement left breast with synthetic substitute, open approach
ICD-10	0HUU0KZ	Supplement left breast with nonautologous tissue substitute, open approach
ICD-10	0HUU37Z	Supplement left breast with autologous tissue substitute, percutaneous approach
ICD-10	0HUU3JZ	Supplement left breast with synthetic substitute, percutaneous approach
ICD-10	0HUU3KZ	Supplement left breast with nonautologous tissue substitute, percutaneous approach
ICD-10	0HUU77Z	Supplement left breast with autologous tissue substitute, via natural or artificial opening
ICD-10	0HUU7JZ	Supplement left breast with synthetic substitute, via natural or artificial opening
ICD-10	0HUU7KZ	Supplement left breast with nonautologous tissue substitute, via natural or artificial opening
ICD-10	0HUU87Z	Supplement left breast with autologous tissue substitute, via natural or artificial opening endoscopic
ICD-10	0HUU8JZ	Supplement left breast with synthetic substitute, via natural or artificial opening endoscopic
ICD-10	0HUU8KZ	Supplement left breast with nonautologous tissue substitute, via natural or artificial opening endoscopic
ICD-10	0HUUX7Z	Supplement left breast with autologous tissue substitute, external approach
ICD-10	0HUUXJZ	Supplement left breast with synthetic substitute, external approach
ICD-10	0HUUXKZ	Supplement left breast with nonautologous tissue substitute, external approach
ICD-10	0HUV07Z	Supplement bilateral breast with autologous tissue substitute, open approach
ICD-10	0HUV0JZ	Supplement bilateral breast with synthetic substitute, open approach
ICD-10	0HUV0KZ	Supplement bilateral breast with nonautologous tissue substitute, open approach
ICD-10	0HUV37Z	Supplement bilateral breast with autologous tissue substitute, percutaneous approach
ICD-10	0HUV3JZ	Supplement bilateral breast with synthetic substitute, percutaneous approach
ICD-10	0HUV3KZ	Supplement bilateral breast with nonautologous tissue substitute, percutaneous approach
ICD-10	0HUV77Z	Supplement bilateral breast with autologous tissue substitute, via natural or artificial opening
ICD-10	0HUV7JZ	Supplement bilateral breast with synthetic substitute, via natural or artificial opening
ICD-10	0HUV7KZ	Supplement bilateral breast with nonautologous tissue substitute, via natural or artificial opening
ICD-10	0HUV87Z	Supplement bilateral breast with autologous tissue substitute, via natural or artificial opening endoscopic
ICD-10	0HUV8JZ	Supplement bilateral breast with synthetic substitute, via natural or artificial opening endoscopic
ICD-10	0HUV8KZ	Supplement bilateral breast with nonautologous tissue substitute, via natural or artificial opening endoscopic
ICD-10	0HUVX7Z	Supplement bilateral breast with autologous tissue substitute, external approach
ICD-10	0HUVXJZ	Supplement bilateral breast with synthetic substitute, external approach
ICD-10	0HUVXKZ	Supplement bilateral breast with nonautologous tissue substitute, external approach
ICD-10	0HUW07Z	Supplement right nipple with autologous tissue substitute, open approach
ICD-10	0HUW0JZ	Supplement right nipple with synthetic substitute, open approach
ICD-10	0HUW0KZ	Supplement right nipple with nonautologous tissue substitute, open approach
ICD-10	0HUW37Z	Supplement right nipple with autologous tissue substitute, percutaneous approach
ICD-10	0HUW3JZ	Supplement right nipple with synthetic substitute, percutaneous approach
ICD-10	0HUW3KZ	Supplement right nipple with nonautologous tissue substitute, percutaneous approach
ICD-10	0HUW77Z	Supplement right nipple with autologous tissue substitute, via natural or artificial opening
ICD-10	0HUW7JZ	Supplement right nipple with synthetic substitute, via natural or artificial opening

© 2025 Balkan E et al. *JAMA Network Open.*

ICD-10	0HUW7KZ	Supplement right nipple with nonautologous tissue substitute, via natural or artificial opening
ICD-10	0HUW87Z	Supplement right nipple with autologous tissue substitute, via natural or artificial opening endoscopic
ICD-10	0HUW8JZ	Supplement right nipple with synthetic substitute, via natural or artificial opening endoscopic
ICD-10	0HUW8KZ	Supplement right nipple with nonautologous tissue substitute, via natural or artificial opening endoscopic
ICD-10	0HUWX7Z	Supplement right nipple with autologous tissue substitute, external approach
ICD-10	0HUWXJZ	Supplement right nipple with synthetic substitute, external approach
ICD-10	0HUWXKZ	Supplement right nipple with nonautologous tissue substitute, external approach
ICD-10	0HUX07Z	Supplement left nipple with autologous tissue substitute, open approach
ICD-10	0HUX0JZ	Supplement left nipple with synthetic substitute, open approach
ICD-10	0HUX0KZ	Supplement left nipple with nonautologous tissue substitute, open approach
ICD-10	0HUX37Z	Supplement left nipple with autologous tissue substitute, percutaneous approach
ICD-10	0HUX3JZ	Supplement left nipple with synthetic substitute, percutaneous approach
ICD-10	0HUX3KZ	Supplement left nipple with nonautologous tissue substitute, percutaneous approach
ICD-10	0HUX77Z	Supplement left nipple with autologous tissue substitute, via natural or artificial opening
ICD-10	0HUX7JZ	Supplement left nipple with synthetic substitute, via natural or artificial opening
ICD-10	0HUX7KZ	Supplement left nipple with nonautologous tissue substitute, via natural or artificial opening
ICD-10	0HUX87Z	Supplement left nipple with autologous tissue substitute, via natural or artificial opening endoscopic
ICD-10	0HUX8JZ	Supplement left nipple with synthetic substitute, via natural or artificial opening endoscopic
ICD-10	0HUX8KZ	Supplement left nipple with nonautologous tissue substitute, via natural or artificial opening endoscopic
ICD-10	0HUXX7Z	Supplement left nipple with autologous tissue substitute, external approach
ICD-10	0HUXXJZ	Supplement left nipple with synthetic substitute, external approach
ICD-10	0HUXXKZ	Supplement left nipple with nonautologous tissue substitute, external approach
ICD-10	0TQD0ZZ	Repair urethra, open approach
ICD-10	0TQD3Z	Repair urethra, percutaneous approach
ICD-10	0TQD4ZZ	Repair urethra, percutaneous endoscopic approach
ICD-10	0TQD8ZZ	Repair urethra, via natural or artificial opening endoscopic
ICD-10	0TQDXZZ	Repair urethra, external approach
ICD-10	0TRD07Z	Replacement of urethra with autologous tissue substitute, open approach
ICD-10	0TRD0JZ	Replacement of urethra with synthetic substitute, open approach
ICD-10	0TRD0KZ	Replacement of urethra with nonautologous tissue substitute, open approach
ICD-10	0TRD47Z	Replacement of urethra with autologous tissue substitute, percutaneous endoscopic approach
ICD-10	0TRD4JZ	Replacement of urethra with synthetic substitute, percutaneous endoscopic approach
ICD-10	0TRD4KZ	Replacement of urethra with nonautologous tissue substitute, percutaneous endoscopic approach
ICD-10	0TRD77Z	Replacement of urethra with autologous tissue substitute, via natural or artificial opening
ICD-10	0TRD7JZ	Replacement of urethra with synthetic substitute, via natural or artificial opening
ICD-10	0TRD7KZ	Replacement of urethra with nonautologous tissue substitute, via natural or artificial opening
ICD-10	0TRD87Z	Replacement of urethra with autologous tissue substitute, via natural or artificial opening endoscopic
ICD-10	0TRD8JZ	Replacement of urethra with synthetic substitute, via natural or artificial opening endoscopic
ICD-10	0TRD8KZ	Replacement of urethra with nonautologous tissue substitute, via natural or artificial opening endoscopic
ICD-10	0TRDX7Z	Replacement of urethra with autologous tissue substitute, external approach
ICD-10	0TRDXJZ	Replacement of urethra with synthetic substitute, external approach

© 2025 Balkan E et al. *JAMA Network Open.*

ICD-10	0TRDXKZ	Replacement of urethra with nonautologous tissue substitute, external approach
ICD-10	0TUD07Z	Supplement urethra with autologous tissue substitute, open approach
ICD-10	0TUD0JZ	Supplement urethra with synthetic substitute, open approach
ICD-10	0TUD0KZ	Supplement urethra with nonautologous tissue substitute, open approach
ICD-10	0TUD47Z	Supplement urethra with autologous tissue substitute, percutaneous endoscopic approach
ICD-10	0TUD4KZ	Supplement urethra with nonautologous tissue substitute, percutaneous endoscopic approach
ICD-10	0TUD77Z	Supplement urethra with autologous tissue substitute, via natural or artificial opening
ICD-10	0TUD7JZ	Supplement urethra with synthetic substitute, via natural or artificial opening
ICD-10	0TUD7KZ	Supplement urethra with nonautologous tissue substitute, via natural or artificial opening
ICD-10	0TUD87Z	Supplement urethra with autologous tissue substitute, via natural or artificial opening endoscopic
ICD-10	0TUD8JZ	Supplement urethra with synthetic substitute, via natural or artificial opening endoscopic
ICD-10	0TUD8KZ	Supplement urethra with nonautologous tissue substitute, via natural or artificial opening endoscopic
ICD-10	0TUDX7Z	Supplement urethra with autologous tissue substitute, external approach
ICD-10	0TUDXJZ	Supplement urethra with synthetic substitute, external approach
ICD-10	0TUDXKZ	Supplement urethra with nonautologous tissue substitute, external approach
ICD-10	0U5J0ZZ	Destruction of clitoris, open approach
ICD-10	0U5JXZZ	Destruction of clitoris, external approach
ICD-10	0U9J00Z	Drainage of clitoris with drainage device, open approach
ICD-10	0U9J0ZZ	Drainage of clitoris, open approach
ICD-10	0U9JX0Z	Drainage of clitoris with drainage device, external approach
ICD-10	0U9JXZZ	Drainage of clitoris, external approach
ICD-10	0UB14ZZ	Excision of left ovary, percutaneous endoscopic approach
ICD-10	0UB20ZZ	Excision of bilateral ovaries, open approach
ICD-10	0UB24ZZ	Excision of bilateral ovaries, percutaneous endoscopic approach
ICD-10	0UB70ZZ	Excision of bilateral fallopian tubes, open approach
ICD-10	0UB74ZZ	Excision of bilateral fallopian tubes, percutaneous endoscopic approach
ICD-10	0UBG0ZZ	Excision of vagina, open approach
ICD-10	0UBG4ZZ	Excision of vagina, percutaneous endoscopic approach
ICD-10	0UBJ0ZZ	Excision of clitoris, open approach
ICD-10	0UBJXZZ	Excision of clitoris, external approach
ICD-10	0UBM0ZZ	Excision of vulva, open approach
ICD-10	0UBMXZZ	Excision of vulva, external approach
ICD-10	0UCJ0ZZ	Extirpation of matter from clitoris, open approach
ICD-10	0UCJXZZ	Extirpation of matter from clitoris, external approach
ICD-10	0ULG7ZZ	Occlusion of vagina, via natural or artificial opening
ICD-10	0UMJXZZ	Reattachment of clitoris, external approach
ICD-10	0UNG7ZZ	Release vagina, via natural or artificial opening
ICD-10	0UNJ0ZZ	Release clitoris, open approach
ICD-10	0UNJXZZ	Release clitoris, external approach
ICD-10	0UQG0ZZ	Repair vagina, open approach
ICD-10	0UQG0ZZ	Repair vagina, open approach
ICD-10	0UQG7ZZ	Repair vagina, via natural or artificial opening
ICD-10	0UQGXZZ	Repair vagina, external approach
ICD-10	0UQJ0ZZ	Repair clitoris, open approach
ICD-10	0UQJXZZ	Repair clitoris, external approach
ICD-10	0UQMXZZ	Repair vulva, external approach
ICD-10	0USG4ZZ	Reposition vagina, percutaneous endoscopic approach
ICD-10	0UT00ZZ	Resection of right ovary, open approach
ICD-10	0UT04ZZ	Resection of right ovary, percutaneous endoscopic approach
ICD-10	0UT07ZZ	Resection of right ovary, via natural or artificial opening
ICD-10	0UT08ZZ	Resection of right ovary, via natural or artificial opening endoscopic

ICD-10	0UT0FZZ	Resection of right ovary, via natural or artificial opening with percutaneous endoscopic assistance
ICD-10	0UT10ZZ	Resection of left ovary, open approach
ICD-10	0UT14ZZ	Resection of left ovary, percutaneous endoscopic approach
ICD-10	0UT17ZZ	Resection of left ovary, via natural or artificial opening
ICD-10	0UT18ZZ	Resection of left ovary, via natural or artificial opening endoscopic
ICD-10	0UT1FZZ	Resection of left ovary, via natural or artificial opening with percutaneous endoscopic assistance
ICD-10	0UT20ZZ	Resection of bilateral ovaries, open approach
ICD-10	0UT24ZZ	Resection of bilateral ovaries, percutaneous endoscopic approach
ICD-10	0UT27ZZ	Resection of bilateral ovaries, via natural or artificial opening
ICD-10	0UT28ZZ	Resection of bilateral ovaries, via natural or artificial opening endoscopic
ICD-10	0UT2FZZ	Resection of bilateral ovaries, via natural or artificial opening with percutaneous endoscopic assistance
ICD-10	0UT40ZZ	Resection of uterine supporting structure, open approach
ICD-10	0UT44ZZ	Resection of uterine supporting structure, percutaneous endoscopic approach
ICD-10	0UT47ZZ	Resection of uterine supporting structure, via natural or artificial opening
ICD-10	0UT48ZZ	Resection of uterine supporting structure, via natural or artificial opening endoscopic
ICD-10	0UT50ZZ	Resection of right fallopian tube, open approach
ICD-10	0UT54ZZ	Resection of right fallopian tube, percutaneous endoscopic approach
ICD-10	0UT60ZZ	Resection of left fallopian tube, open approach
ICD-10	0UT64ZZ	Resection of left fallopian tube, percutaneous endoscopic approach
ICD-10	0UT6FZZ	Resection of left fallopian tube, via natural or artificial opening with percutaneous endoscopic assistance
ICD-10	0UT70ZZ	Resection of bilateral fallopian tubes, open approach
ICD-10	0UT74ZZ	Resection of bilateral fallopian tubes, percutaneous endoscopic approach
ICD-10	0UT77ZZ	Resection of bilateral fallopian tubes, via natural or artificial opening
ICD-10	0UT78ZZ	Resection of bilateral fallopian tubes, via natural or artificial opening endoscopic
ICD-10	0UT7FZZ	Resection of bilateral fallopian tubes, via natural or artificial opening with percutaneous endoscopic assistance
ICD-10	0UT90ZL	Resection of uterus, supracervical, open approach
ICD-10	0UT90ZZ	Resection of uterus, open approach
ICD-10	0UT92ZL	Resection of uterus, supracervical
ICD-10	0UT94ZL	Resection of uterus, supracervical, percutaneous endoscopic approach
ICD-10	0UT94ZZ	Resection of uterus, percutaneous endoscopic approach
ICD-10	0UT94ZZ	Resection of uterus, percutaneous endoscopic approach
ICD-10	0UT97ZL	Resection of uterus, supracervical, via natural or artificial opening
ICD-10	0UT97ZZ	Resection of uterus, via natural or artificial opening
ICD-10	0UT98ZL	Resection of uterus, supracervical, via natural or artificial opening endoscopic
ICD-10	0UT98ZZ	Resection of uterus, via natural or artificial opening endoscopic
ICD-10	0UT9FZL	Resection of uterus, supracervical, via natural or artificial opening with percutaneous endoscopic assistance
ICD-10	0UT9FZZ	Resection of uterus, via natural or artificial opening with percutaneous endoscopic assistance
ICD-10	0UTC0ZZ	Resection of cervix, open approach
ICD-10	0UTC0ZZ	Resection of cervix, open approach
ICD-10	0UTC4ZZ	Resection of cervix, percutaneous endoscopic approach
ICD-10	0UTC7ZZ	Resection of cervix, via natural or artificial opening
ICD-10	0UTC8ZZ	Resection of cervix, via natural or artificial opening
ICD-10	0UTG0ZZ	Resection of vagina, open approach
ICD-10	0UTG4ZZ	Resection of vagina, percutaneous endoscopic approach
ICD-10	0UTG7ZZ	Resection of vagina, via natural or artificial opening
ICD-10	0UTG8ZZ	Resection of vagina, via natural or artificial opening endoscopic
ICD-10	0UTJ0ZZ	Resection of clitoris, open approach
ICD-10	0UTJXZZ	Resection of clitoris, external approach
ICD-10	0UTMXZZ	Resection of vulva, external approach
ICD-10	0UUG07Z	Supplement vagina with autologous tissue substitute, open approach
ICD-10	0UUG07Z	Supplement vagina with autologous tissue substitute, open approach

© 2025 Balkan E et al. *JAMA Network Open.*

ICD-10	0UUG0JZ	Supplement vagina with synthetic substitute, open approach
ICD-10	0UUG0JZ	Supplement vagina with synthetic substitute, open approach
ICD-10	0UUG0KZ	Supplement vagina with nonautologous tissue substitute, open approach
ICD-10	0UUG0KZ	Supplement vagina with nonautologous tissue substitute, open approach
ICD-10	0UUG47Z	Supplement vagina with autologous tissue substitute, percutaneous endoscopic approach
ICD-10	0UUG47Z	Supplement vagina with autologous tissue substitute, percutaneous endoscopic approach
ICD-10	0UUG4JZ	Supplement vagina with synthetic substitute, percutaneous endoscopic approach
ICD-10	0UUG4JZ	Supplement vagina with synthetic substitute, percutaneous endoscopic approach
ICD-10	0UUG4KZ	Supplement vagina with nonautologous tissue substitute, percutaneous endoscopic approach
ICD-10	0UUG4KZ	Supplement vagina with nonautologous tissue substitute, percutaneous endoscopic approach
ICD-10	0UUG77Z	Supplement vagina with autologous tissue substitute, via natural or artificial opening
ICD-10	0UUG77Z	Supplement vagina with autologous tissue substitute, via natural or artificial opening
ICD-10	0UUG7JZ	Supplement vagina with synthetic substitute, via natural or artificial opening
ICD-10	0UUG7JZ	Supplement vagina with synthetic substitute, via natural or artificial opening
ICD-10	0UUG7KZ	Supplement vagina with nonautologous tissue substitute, via natural or artificial opening
ICD-10	0UUG7KZ	Supplement vagina with nonautologous tissue substitute, via natural or artificial opening
ICD-10	0UUG87Z	Supplement vagina with autologous tissue substitute, via natural or artificial opening endoscopic
ICD-10	0UUG87Z	Supplement vagina with autologous tissue substitute, via natural or artificial opening endoscopic
ICD-10	0UUG8JZ	Supplement vagina with synthetic substitute, via natural or artificial opening endoscopic
ICD-10	0UUG8JZ	Supplement vagina with synthetic substitute, via natural or artificial opening endoscopic
ICD-10	0UUG8KZ	Supplement vagina with nonautologous tissue substitute, via natural or artificial opening endoscopic
ICD-10	0UUG8KZ	Supplement vagina with nonautologous tissue substitute, via natural or artificial opening endoscopic
ICD-10	0UUGX7Z	Supplement vagina with autologous tissue substitute, external approach
ICD-10	0UUGX7Z	Supplement vagina with autologous tissue substitute, external approach
ICD-10	0UUGXJZ	Supplement vagina with synthetic substitute, external approach
ICD-10	0UUGXJZ	Supplement vagina with synthetic substitute, external approach
ICD-10	0UUGXKZ	Supplement vagina with nonautologous tissue substitute, external approach
ICD-10	0UUGXKZ	Supplement vagina with nonautologous tissue substitute, external approach
ICD-10	0UUJ07Z	Supplement clitoris with autologous tissue substitute, open approach
ICD-10	0UUJ0JZ	Supplement clitoris with synthetic substitute, open approach
ICD-10	0UUJ0KZ	Supplement clitoris with nonautologous tissue substitute, open approach
ICD-10	0UUJX7Z	Supplement clitoris with autologous tissue substitute, external approach
ICD-10	0UUJXJZ	Supplement clitoris with synthetic substitute, external approach
ICD-10	0UUJXKZ	Supplement clitoris with nonautologous tissue substitute, external approach
ICD-10	0UUM07Z	Supplement vulva with autologous tissue substitute, open approach
ICD-10	0UWH47Z	Revision of autologous tissue substitute in vagina and cul-de-sac, percutaneous endoscopic approach
ICD-10	0UWH77Z	Revision of autologous tissue substitute in vagina and cul-de-sac, via natural or artificial opening
ICD-10	0V590ZZ	Destruction of right testis, open approach
ICD-10	0V593ZZ	Destruction of right testis, percutaneous approach
ICD-10	0V594ZZ	Destruction of right testis, percutaneous endoscopic approach
ICD-10	0V5B0ZZ	Destruction of left testis, open approach
ICD-10	0V5B3ZZ	Destruction of left testis, percutaneous approach
ICD-10	0V5B4ZZ	Destruction of left testis, percutaneous endoscopic approach
ICD-10	0V5C0ZZ	Destruction of bilateral testes, open approach

ICD-10	0V5C3ZZ	Destruction of bilateral testes, percutaneous approach
ICD-10	0V5C4ZZ	Destruction of bilateral testes, percutaneous endoscopic approach
ICD-10	0VB50ZZ	Excision of scrotum, open approach
ICD-10	0VB5XZZ	Excision of scrotum, external approach
ICD-10	0VB90ZZ	Excision of right testis, open approach
ICD-10	0VB93ZZ	Excision of right testis, percutaneous approach
ICD-10	0VB94ZZ	Excision of right testis, percutaneous endoscopic approach
ICD-10	0VBB0ZZ	Excision of left testis, open approach
ICD-10	0VBB3ZZ	Excision of left testis, percutaneous approach
ICD-10	0VBB4ZZ	Excision of left testis, percutaneous endoscopic approach
ICD-10	0VBC0ZZ	Excision of bilateral testes, open approach
ICD-10	0VBC3ZZ	Excision of bilateral testes, percutaneous approach
ICD-10	0VBC4ZZ	Excision of bilateral testes, percutaneous endoscopic approach
ICD-10	0VBS0ZZ	Excision of penis, open approach
ICD-10	0VHD0YZ	Insertion of other device into testis, open approach
ICD-10	0VPS0JZ	Removal of synthetic substitute from penis, open approach
ICD-10	0VQ50ZZ	Repair scrotum, open approach
ICD-10	0VQ5XZZ	Repair scrotum, external approach
ICD-10	0VR90JZ	Replacement of right testis with synthetic substitute, open approach
ICD-10	0VRB0JZ	Replacement of left testis with synthetic substitute, open approach
ICD-10	0VRC0JZ	Replacement of bilateral testes with synthetic substitute, open approach
ICD-10	0VT50ZZ	Resection of scrotum, open approach
ICD-10	0VT90ZZ	Resection of right testis, open approach
ICD-10	0VT94ZZ	Resection of right testis, percutaneous endoscopic approach
ICD-10	0VTB0ZZ	Resection of left testis, open approach
ICD-10	0VTB4ZZ	Resection of left testis, percutaneous endoscopic approach
ICD-10	0VTC0ZZ	Resection of bilateral testes, open approach
ICD-10	0VTC4ZZ	Resection of bilateral testes, percutaneous endoscopic approach
ICD-10	0VTS0ZZ	Resection of penis, open approach
ICD-10	0VTS4ZZ	Resection of penis, percutaneous endoscopic approach
ICD-10	0VTSXZZ	Resection of penis, external approach
ICD-10	0VU5X7Z	Supplement scrotum with autologous tissue substitute, external approach
ICD-10	0VU907Z	Supplement right testis with autologous tissue substitute, open approach
ICD-10	0VU90JZ	Supplement right testis with synthetic substitute, open approach
ICD-10	0VU90KZ	Supplement right testis with nonautologous tissue substitute, open approach
ICD-10	0VUB07Z	Supplement left testis with autologous tissue substitute, open approach
ICD-10	0VUB0JZ	Supplement left testis with synthetic substitute, open approach
ICD-10	0VUB0KZ	Supplement left testis with nonautologous tissue substitute, open approach
ICD-10	0VUC07Z	Supplement bilateral testes with autologous tissue substitute, open approach
ICD-10	0VUC0JZ	Supplement bilateral testes with synthetic substitute, open approach
ICD-10	0VUC0KZ	Supplement bilateral testes with nonautologous tissue substitute, open approach
ICD-10	0VUS072	Supplement penis with autologous tissue substitute, open approach
ICD-10	0VUS07Z	Supplement penis with autologous tissue substitute
ICD-10	0VUS07Z	Supplement penis with autologous tissue substitute, open approach
ICD-10	0VUS0JZ	Supplement penis with synthetic substitute
ICD-10	0VUS0JZ	Supplement penis with synthetic substitute, open approach
ICD-10	0VUS0JZ	Supplement penis with synthetic substitute, open approach
ICD-10	0VUS0KZ	Supplement penis with nonautologous tissue substitute
ICD-10	0VUS0KZ	Supplement penis with nonautologous tissue substitute, open approach
ICD-10	0VUS0KZ	Supplement penis with nonautologous tissue substitute, open approach
ICD-10	0VUS47Z	Supplement penis with autologous tissue substitute
ICD-10	0VUS47Z	Supplement penis with autologous tissue substitute, percutaneous endoscopic approach
ICD-10	0VUS47Z	Supplement penis with autologous tissue substitute, percutaneous endoscopic approach
ICD-10	0VUS4JZ	Supplement penis with synthetic substitute, percutaneous endoscopic approach
ICD-10	0VUS4JZ	Supplement penis with synthetic substitute, percutaneous endoscopic approach
ICD-10	0VUS4JZ	Supplement penis with synthetic substitute, percutaneous endoscopic approach

© 2025 Balkan E et al. *JAMA Network Open.*

ICD-10	0VUS4KZ	Supplement penis with nonautologous tissue substitute
ICD-10	0VUS4KZ	Supplement penis with nonautologous tissue substitute, percutaneous endoscopic approach
ICD-10	0VUS4KZ	Supplement penis with nonautologous tissue substitute, percutaneous endoscopic approach
ICD-10	0VUSX7Z	Supplement penis with autologous tissue substitute
ICD-10	0VUSXJZ	Supplement penis with synthetic substitute
ICD-10	0VUSXKZ	Supplement penis with nonautologous tissue substitute
ICD-10	0W4M070	Creation of vagina in male perineum with autologous tissue substitute, open approach.
ICD-10	0W4M0J0	Creation of vagina in male perineum with synthetic substitute, open approach
ICD-10	0W4M0K0	Creation of vagina in male perineum with nonautologous tissue substitute, open approach
ICD-10	0W4M0Z0	Creation of vagina in male perineum, open approach
ICD-10	0W4N071	Creation of penis in female perineum with autologous tissue substitute, open approach
ICD-10	0W4N0J1	Creation of penis in female perineum with synthetic substitute, open approach
ICD-10	0W4N0K1	Creation of penis in female perineum with nonautologous tissue substitute, open approach
ICD-10	0W4N0Z1	Creation of penis in female perineum, open approach
ICD-10	0WPN0JZ	Removal of synthetic substitute from female perineum, open approach
ICD-10	0WUN07Z	Supplement female perineum with autologous tissue substitute, open approach
ICD-10	OHRT0JZ	Replacement of right breast with synthetic substitute, open approach
ICD-10	OHRT0KZ	Replacement of right breast with nonautologous tissue substitute, open approach
ICD-10	T83090A	Other mechanical complication of cystostomy catheter, initial encounter
ICD-10	T83092A	Other mechanical complication of nephrostomy catheter, initial encounter
ICD-10	T83098A	Other mechanical complication of urinary stent, initial encounter
ICD-10	T83193A	Other mechanical complication of other urinary devices and implants, initial encounter
ICD-10	T8329XA	Other mechanical complication of graft of urinary organ, initial encounter
ICD-10	T83410A	Breakdown (mechanical) of implanted penile prosthesis, initial encounter
ICD-10	T83420A	Displacement of implanted penile prosthesis, initial encounter
ICD-10	T83490A	Other mechanical complication of implanted penile prosthesis, initial encounter
ICD-10	T83498A	Other mechanical complication of other prosthetic devices, implants and grafts of genital tract, initial encounter
ICD-10	Z44.30	Encounter for fitting and adjustment of external breast prosthesis, unspecified breast
ICD-10	Z4430	Encounter for fitting and adjustment of external breast prothesis, unspecified breast
ICD-10	Z9071	Acquired absence of both cervix and uterus
ICD-10	Z9072	Acquired absence of ovaries
ICD-9	71.4	Operations on clitoris
ICD-9	85.86	Transposition of nipple
ICD-9	85.87	Other repair of nipple
ICD-9	623	Unilateral orchiectomy
ICD-9	627	Insertion of testicular prosthesis
ICD-9	643	Amputation of penis
ICD-9	645	Operations for sex transformation, not elsewhere classified
ICD-9	674	Amputation of cervix
ICD-9	683	Subtotal abdominal hysterectomy
ICD-9	684	Total abdominal hysterectomy
ICD-9	685	Vaginal hysterectomy
ICD-9	686	Radical abdominal hysterectomy
ICD-9	687	Radical vaginal hysterectomy
ICD-9	689	Other and unspecified hysterectomy
ICD-9	704	Obliteration and total excision of vagina
ICD-9	704	Obliteration and total excision of vagina
ICD-9	706	Vaginal construction
ICD-9	708	Obliteration of vaginal vault
ICD-9	852	Excision or destruction of breast tissue not otherwise specified

© 2025 Balkan E et al. *JAMA Network Open.*

ICD-9	855	Augmentation mammoplasty not otherwise specified
ICD-9	6149	Other repair of scrotum and tunica vaginalis
ICD-9	6241	Bilateral orchiectomy
ICD-9	6443	Construction of penis
ICD-9	6444	Reconstruction of penis
ICD-9	6449	Other repair of penis
ICD-9	6495	Insertion or replacement of non-inflatable penile prosthesis
ICD-9	6497	Insertion or replacement of inflatable penile prosthesis
ICD-9	6501	Laparoscopic oophorotomy
ICD-9	6509	Other oophorotomy
ICD-9	6531	Laparoscopic unilateral oophorectomy
ICD-9	6539	Other unilateral oophorotomy
ICD-9	6541	Laparoscopic unilateral salpingo-oophorotomy
ICD-9	6549	Other laparoscopic unilateral salpingo-oophorotomy
ICD-9	6551	Other removal both ovaries at same operative episode
ICD-9	6552	Other removal of remaining ovary
ICD-9	6553	Lap removal both ovaries at same operative episode
ICD-9	6554	Lap removal remaining ovary
ICD-9	6561	Bilateral salpingo-oophorectomy
ICD-9	6562	Other removal remaining ovary and tube
ICD-9	6563	Bilateral salpingo-oophorectomy
ICD-9	6564	Lap removal remaining ovary and tube
ICD-9	6651	Total bilateral salpingectomy
ICD-9	6831	Laparoscopic supracervical hyserectomy
ICD-9	6839	Other and unspecified subtotal abdominal hysterectomy
ICD-9	6841	Laparoscopic total abdominal hysterectomy
ICD-9	6849	Other and unspecified total abdominal hysterectomy
ICD-9	6851	Laparoscopically assisted vaginal hysterectomy
ICD-9	6859	Other and unspecified vaginal hysterectomy
ICD-9	6861	Laparoscopic radical abdominal hysterectomy
ICD-9	6869	Other and unspecified radical abdominal hysterectomy
ICD-9	6871	Laparoscopic radical vaginal hysterectomy
ICD-9	6879	Other and unspecified radical vaginal hysterectomy
ICD-9	7061	Vaginal construction
ICD-9	7062	Vaginal reconstruction
ICD-9	7063	Vaginal construction with graft or prosthesis
ICD-9	7064	Vaginal reconstruction with graft or prosthesis
ICD-9	7162	Vulvectomy / bilateral vulvectomy
ICD-9	7162	Bilateral vulvectomy
ICD-9	8523	Subtotal mastectomy
ICD-9	8531	Unilateral reduction mammoplasty
ICD-9	8532	Bilateral reduction mammoplasty
ICD-9	8534	Other unilateral subcutaneous mammectomy
ICD-9	8536	Other bilateral subcutaneous mammectomy
ICD-9	8541	Unilateral simple mastectomy
ICD-9	8542	Bilateral mastectomy
ICD-9	8543	Unilateral extended simple mastectomy
ICD-9	8544	Bilateral mastectomy
ICD-9	8545	Unilateral radical mastectomy
ICD-9	8546	Bilateral mastectomy
ICD-9	8547	Unilateral extended radical mastectomy
ICD-9	8548	Bilateral extended mastectomy
ICD-9	8550	Augmentation mammoplasty, not otherwise specified
ICD-9	8551	Unilateral injection into breast for augmentation
ICD-9	8552	Bilateral injection into breast for augmentation
ICD-9	8553	Unilateral breast implant
ICD-9	8554	Bilateral breast implant
ICD-9	8555	Fat graft to breast

ICD-9	8559	Other mammoplasty
ICD-9	8589	Other mammoplasty
ICD-9	8595	Insertion of breast tissue expander
ICD-9	8596	Removal of breast tissue expander
ICD-9	99639	Breakdown (mechanical) of implanted penile prosthesis, initial encounter
ICD-9	V524	Fitting and adjustment of breast prosthesis and implant

© 2025 Balkan E et al. *JAMA Network Open.*

eTable 2. Cancer-Related Diagnosis Codes That Exclude Transgender and Gender Diverse Individuals from Our Analysis if Their Surgery Could Be Cancer-Related Care

Cancer type	ICD-9 or -10	Code	Code description
Uterine	ICD-9	183.8	Malignant neoplasm of other specified sites of uterine adnexa
	ICD-9	183.9	Malignant neoplasm of uterine adnexa, unspecified site
	ICD-10	C57.4	Malignant neoplasm of uterine adnexa, unspecified
	ICD-9	183	Malignant neoplasm of ovary and other uterine adnexa
Ovarian	ICD-9	183	Malignant neoplasm of ovary and other uterine adnexa
	ICD-10	C56	Malignant neoplasm of the ovary
Cervical	ICD-9	180.1	Malignant neoplasm of exocervix
	ICD-9	180.8	Malignant neoplasm of other specified sites of cervix
	ICD-9	180.9	Malignant neoplasm of cervix uteri, unspecified site
	ICD-9	184	Malignant neoplasm of other and unspecified female genital organs
	ICD-10	C53.0	Malignant neoplasm of endocervix
	ICD-10	C53.1	Malignant neoplasm of exocervix
	ICD-10	C53.8	Malignant neoplasm of overlapping sites of cervix uteri
	ICD-10	C53.9	Malignant neoplasm of cervix uteri, unspecified
	ICD-10-CM	Z85.41	Personal history of malignant neoplasm of cervix uteri
Other	ICD-9	183.2	Malignant neoplasm of unspecified fallopian tube
	ICD-10	C57.0	Malignant neoplasm of fallopian tube
	ICD-10	C57.7	Malignant neoplasm of other specified female genital organs
	ICD-10	C57.8	Malignant neoplasm of overlapping sites of female genital organs
	ICD-10	C57.9	Malignant neoplasm of female genital organ, unspecified
Vaginal	ICD-9	184	Malignant neoplasm of other and unspecified female genital organs
	ICD-10	C51	Malignant neoplasm of vulva
	ICD-10	C52	Malignant neoplasm of vagina
Testicular	ICD-9	186	Malignant neoplasm of testis
	ICD-9	187.5	Malignant neoplasm of unspecified epididymis
	ICD-9	187.6	Malignant neoplasm of unspecified spermatic cord
	ICD-9	187.7	Malignant neoplasm of scrotum
	ICD-9	187.8	Malignant neoplasm of overlapping sites of penis
	ICD-9	187.9	Malignant neoplasm of male genital organ, site unspecified
	ICD-10	C62	Malignant neoplasm of testis
	ICD-10	C63	Malignant neoplasm of other and unspecified male genital organs
Penile	ICD-9	187.1	Malignant neoplasm of prepuce
	ICD-9	187.2	Malignant neoplasm of glans penis
	ICD-9	187.3	Malignant neoplasm of body of penis
	ICD-9	187.4	Malignant neoplasm of penis, unspecified.
	ICD-10	C60	Malignant neoplasm of penis

© 2025 Balkan E et al. *JAMA Network Open.*

eTable 3. Rate of Gender-Affirming Surgery in CMS Regions per 1,000 Beneficiary-Years by Gender Identity, January 2016-February 2020

**CMS Region**	**Rate of GAS, No. (95% CI)**	
	TGD	Non-TGD
1: Connecticut, Maine, Massachusetts, New Hampshire, Rhode Island, Vermont	17.7 (15.1-20.2)	4.7 (3.5-6.0)
2: New Jersey, New York, Puerto Rico, Virgin Islands	14.0 (11.8-16.2)	5.9 (4.8-7.0)
3: Delaware, District of Columbia, Maryland, Pennsylvania, Virginia, West Virginia	14.5 (12.4-16.7)	5.2 (4.1-6.3)
4: Alabama, Florida, Georgia, Kentucky, Mississippi, North Carolina, South Carolina, Tennessee	12.5 (10.9-14.1)	4.3 (3.5-5.2)
5: Illinois, Indiana, Michigan, Minnesota, Ohio, Wisconsin	15.6 (13.9-17.3)	4.6 (3.6-5.5)
6: Arkansas, Louisiana, New Mexico, Oklahoma, Texas	9.9 (8.0-11.8)	4.1 (3.2-5.0)
7: Iowa, Kansas, Missouri, Nebraska	14.6 (11.5-17.6)	4.7 (3.3-6.1)
8: Colorado, Montana, North Dakota, South Dakota, Utah, Wyoming	14.5 (10.9-18.0)	4.5 (3.0-6.0)
9: Arizona, California, Hawaii, Nevada, Pacific territories	18.4 (16.2-20.4)	4.5 (3.6-5.4)
10: Alaska, Idaho, Oregon, Washington	22.8 (19.4-26.3)	5.4 (3.9-6.9)

© 2025 Balkan E et al. *JAMA Network Open.*

eTable 4. Associations between Transgender Beneficiary Characteristics and Gender-Affirming Surgery, Replacing CMS Regions with Medicare Administrative Contractor Regions, January 2016-February 2020

Category		Odds ratio (95% CI)	p-value
Medicare-Medicaid dual		1.12 (1.01-1.25)	0.03
Age Category	18-30	1.61 (1.29-2.01)	0.000
Reference Group = 61-65	31-40	1.94 (1.58-2.39)	0.000
	41-50	1.77 (1.44-2.18)	0.000
	51-55	1.51 (1.20-1.91)	0.000
	56-60	1.25 (0.99-1.59)	0.06
	66-70	0.80 (0.65-0.98)	0.03
	71-75	0.61 (0.49-0.78)	0.000
	over 75	0.39 (0.31-0.49)	0.000
Race or Ethnicity	American Indian or Alaska Native	0.78 (0.45-1.33)	0.36
Reference Group = Black (or African American)	Asian or Pacific Islander	0.80 (0.54-1.18)	0.26
	Hispanic	0.95 (0.76-1.18)	0.64
	Non-Hispanic White	0.95 (0.82-1.09)	0.45
	unknown	0.75 (0.52-1.09)	0.13
	Other*	0.31 (0.12-0.82)	0.02
Original Reason for Medicare entitlement	age	1.40 (1.15-1.69)	0.001
Reference Group = Disability	ESKD or ESKD and disability**	0.87 (0.59-1.28)	0.48
A/B MAC Region	JL: New Jersey, Pennsylvania, Delaware, District of Columbia, Maryland	0.88 (0.73-1.07)	0.20
Reference Group = Jurisdiction (J) K (the Northeast and New York)			
	JM: West Virginia, Virginia, North Carolina, South Carolina	0.59 (0.46-0.75)	0.000
	JJ: Tennessee, Georgia, Alabama	0.65 (0.49-0.85)	0.002
	JN: Florida, Puerto Rico, US Virgin Islands	0.93 (0.74-1.16)	0.52
	J15: Ohio, Kentucky	0.70 (0.54-0.92)	0.01
	J8: Michigan, Indiana	1.05 (0.85-1.29)	0.65
	J6: Minnesota, Wisconsin, Illinois	0.94 (0.77-1.15)	0.55
	J5: Kansas, Iowa, Missouri, Nebraska	0.86 (0.68-1.10)	0.23
	JH: New Mexico, Colorado, Texas, Oklahoma, Louisiana, Mississippi, Arkansas	0.63 (0.51-0.78)	0.000
	JF: Washington, Oregon, Idaho, Wyoming, Montana, North Dakota, South Dakota, Utah, Arizona, Alaska	1.20 (1.00-1.42)	0.045
	JE: California, Nevada, Hawaii, Guam, Northern Mariana Islands, American Samoa	1.11 (0.94-1.32)	0.23
	Other/Living outside the regions	0.64 (0.07-5.59)	0.68
Chronic Conditions: #	1	1.32 (1.06-1.64)	0.01
Reference Group = 0	2-5	1.51 (1.30-1.75)	0.000
	6-9	1.58 (1.35-1.84)	0.000
	10+	2.07 (1.77-2.43)	0.000

© 2025 Balkan E et al. *JAMA Network Open.*

*Medicare's classification of "other" can be interpreted as a non-Hispanic race/ethnicity other than those offered, and "unknown" suggests that the data is missing for an individual.[30]
**ESKD = End Stage Kidney Disease

© 2025 Balkan E et al. *JAMA Network Open.*

eTable 5. Associations between Transgender Beneficiary Characteristics and Gender-Affirming Surgery, Replacing CMS Region with Medicare Administrative Contractors, January 2016-February 2020

Category		Odds ratio (95% CI)	p-value
Medicare-Medicaid dual		1.12 (1.01-1.25)	0.03
Age Category	30 years & under	1.61 (1.29-2.01)	0.000
Reference Group = 61-65	31-40	1.94 (1.58-2.39)	0.000
	41-50	1.77 (1.44-2.18)	0.000
	51-55	1.51 (1.20-1.90)	0.000
	56-60	1.25 (0.99-1.58)	0.06
	66-70	0.80 (0.65-0.98)	0.03
	71-75	0.62 (0.49-0.78)	0.000
	over 75	0.39 (0.31-0.49)	0.000
Race or Ethnicity	American Indian or Alaska Native	0.75 (0.44-1.29)	0.30
Reference Group = Black or African American	Asian or Pacific Islander	0.79 (0.53-1.17)	0.24
	Hispanic	0.92 (0.74-1.15)	0.47
	Non-Hispanic white	0.94 (0.82-1.09)	0.42
	unknown	0.75 (0.52-1.09)	0.13
	Other*	0.30 (0.11-0.80)	0.02
Original Reason for Medicare entitlement	age	1.40 (1.15-1.69)	0.001
Reference Group = Disability	ESKD or ESKD and disability**	0.87 (0.59-1.28)	0.47
Regional MAC Reference Group = National Government Services: the Northeast, New York, Minnesota, Wisconsin, Illinois	Novitas: New Jersey, Pennsylvania, Maryland, Delaware, District of Columbia, Colorado, New Mexico, Texas, Oklahoma, Arkansas, Louisiana, Mississippi	0.76 (0.66-0.88)	0.000
	Palmetto: West Virginia, Virginia, North Carolina, South Carolina, Tennessee, Alabama, Georgia	0.62 (0.51-0.75)	0.000
	First Coast Service Options: Florida, Puerto Rico, US Virgin Islands	0.95 (0.76-1.18)	0.63
	CGS: Ohio, Kentucky	0.71 (0.55-0.93)	0.01
	WPS: Michigan, Indiana, Kansas, Iowa, Missouri, Nebraska	0.98 (0.84-1.16)	0.85
	Noridian: Washington, Oregon, Idaho, Wyoming, Montana, North Dakota, South Dakota, Utah, Arizona, Alaska, California, Nevada, Hawaii, Guam, Northern Mariana Islands, American Samoa	1.17 (1.03-1.34)	0.02
	Other/Living outside the regions	0.65 (0.07-5.73)	0.70
Chronic Conditions: #	1	1.32 (1.06-1.64)	0.01
Reference Group = 0	2-5	1.51 (1.30-1.75)	0.000
	6-9	1.58 (1.35-1.85)	0.000
	10+	2.08 (1.78-2.44)	0.000

© 2025 Balkan E et al. *JAMA Network Open.*

*Medicare's classification of "other" can be interpreted as a non-Hispanic race/ethnicity other than those offered, and "unknown" suggests that the data is missing for an individual.[30]
**ESKD = End Stage Kidney Disease

© 2025 Balkan E et al. *JAMA Network Open.*

eTable 6. Characteristics of Beneficiaries Not Identified as Transgender or Gender Diverse Who Did and Did Not Receive Surgeries That Could Be Classified as Gender-Affirming at the Person-Year Level, January 2016–February 2020

		**No surgery N=159,626 No. (%)**	**Received surgery N=761 No. (%)**	**p-value**
Medicare-Medicaid dually enrolled		69,173 (43.3)	393 (51.6)	<0.001
Age, years	18-30	16,272 (10.2)	79 (10.4)	0.068
	31-40	15,401 (9.6)	89 (11.7)	
	41-50	15,166 (9.5)	96 (12.6)	
	51-55	8,380 (5.2)	37 (4.9)	
	56-60	8,081 (5.1)	38 (5.0)	
	61-65	11,303 (7.1)	48 (6.3)	
	66-70	18,033 (11.3)	79 (10.4)	
	71-75	17,039 (10.7)	71 (9.3)	
	over 75	49,951 (31.3)	224 (29.4)	
Race or Ethnicity	American Indian or Alaska Native	5,897 (3.7)	27 (3.5)	0.002
	Asian or Pacific Islander	12,378 (7.8)	45 (5.9)	
	Black or African American	35,885 (22.5)	211 (27.7)	
	Hispanic	29,239 (18.3)	125 (16.4)	
	Non-Hispanic White	62,521 (39.2)	306 (40.2)	
	Unknown	8,243 (5.2)	32 (4.2)	
	Other*	5,463 (3.4)	15 (2.0)	
Original Reason for Medicare Entitlement	Age	66,150 (41.4)	265 (34.8)	<0.001
	Disability	82,724 (51.8)	406 (53.4)	
	ESKD or ESKD and disability**	10,752 (6.7)	90 (11.8)	
CMS Region	1: Connecticut, Maine, Massachusetts, New Hampshire, Rhode Island, Vermont	11,818 (7.4)	55 (7.2)	0.18
	2: New Jersey, New York, Puerto Rico, Virgin Islands	19,924 (12.5)	116 (15.2)	

© 2025 Balkan E et al. *JAMA Network Open.*

		No surgery N=159,626 No. (%)	Received surgery N=761 No. (%)	p-value
CMS Region	3: Delaware, District of Columbia, Maryland, Pennsylvania, Virginia, West Virginia	15,582 (9.8)	88 (11.6)	
	4: Alabama, Florida, Georgia, Kentucky, Mississippi, North Carolina, South Carolina, Tennessee	20,707 (13.0)	98 (12.9)	
	5: Illinois, Indiana, Michigan, Minnesota, Ohio, Wisconsin	18,666 (11.7)	86 (11.3)	
	6: Arkansas, Louisiana, New Mexico, Oklahoma, Texas	19,313 (12.1)	83 (10.9)	
	7: Iowa, Kansas, Missouri, Nebraska	9,469 (5.9)	46 (6.0)	
	8: Colorado, Montana, North Dakota, South Dakota, Utah, Wyoming	9,296 (5.8)	38 (5.0)	
	9: Arizona, California, Hawaii, Nevada, Pacific Territories	23,465 (14.7)	98 (12.9)	
	10: Alaska, Idaho, Oregon, Washington	10,767 (6.7)	53 (7.0)	
	Other/Living outside the 10 CMS Regions	619 (0.4)	* (0.0)	
# of chronic conditions	0	43,555 (27.3)	110 (14.5)	<0.001
	1	9,475 (5.9)	25 (3.3)	
	2-5	46,101 (28.9)	170 (22.3)	
	6-9	34,962 (21.9)	182 (23.9)	
	10+	25,533 (16.0)	274 (36.0)	

All variables are categorical or binary, and are compared using Pearson's chi-square test.
*Medicare's classification of "other" can be interpreted as a non-Hispanic race/ethnicity other than those offered, and "unknown" suggests that the data is missing for an individual.[30]
**ESKD = End Stage Kidney Disease

© 2025 Balkan E et al. *JAMA Network Open.*

eTable 7. Most Common Codes From eTable 1 Found in Surgery Claims, by Gender Modality

NON-TGD COHORT				TGD COHORT			
**Code type**	**Code**	**Code description**	**% of all GAS codes**	**Code type**	**Code**	**Code description**	**% of all GAS codes**
**ICD-10**	T83098A	Other mechanical complication of urinary stent, initial encounter	29.35	**ICD-10**	T83098A	Other mechanical complication of urinary stent, initial encounter	13.26
**ICD-10**	T83498A	Other mechanical complication of other prosthetic devices, implants and grafts of genital tract, initial encounter	11.33	**ICD-10**	0W4M070	Creation of vagina in male perineum with autologous tissue substitute, open approach	6.85
**ICD-10**	T83090A	Other mechanical complication of cystostomy catheter, initial encounter	4.21	**ICD-10**	T83498A	Other mechanical complication of other prosthetic devices, implants and grafts of genital tract, initial encounter	5.22
**ICD-10**	0UT90ZZ	Resection of uterus, open approach	4.01	**CPT**	54520	Excision procedures on the testis	4.85
**ICD-10**	0UT70ZZ	Resection of bilateral fallopian tubes, open approach	3.94	**CPT**	19303	Mastectomy, simple, complete	4.74
**CPT**	52601	Transurethral electrosurgical resection of prostate, including control of postoperative bleeding, complete vasectomy, meatotomy, cystourethroscopy, urethral calibration and/or dilation, and internal urethrotomy	3.66	**CPT**	58571	Laparoscopy, surgical, with total hysterectomy, for uterus 250 g or less; with removal of tube(s) and/or ovary(s)	3.40
**ICD-10**	0UT20ZZ	Resection of bilateral ovaries, open approach	2.76	**ICD-10**	0VTC0ZZ	Resection of bilateral testes, open approach	3.20
**ICD-10**	0UB70ZZ	Excision of bilateral fallopian tubes, open approach	2.42	**CPT**	58661	Laparoscopy removal of adnexa	2.97
**CPT**	58571	Laparoscopy, surgical, with total hysterectomy, for uterus 250 g or less; with removal of tube(s) and/or ovary(s)	1.80	**CPT**	19325	Mammaplasty, augmentation; with prosthetic implant	2.80
**ICD-10**	T83490A	Other mechanical complication of implanted penile prosthesis, initial encounter	1.73	**ICD-10**	T83090A	Other mechanical complication of cystostomy catheter, initial encounter	2.62

© 2025 Balkan E et al. *JAMA Network Open.*

**ICD-10**	0UTC0ZZ	Resection of cervix, open approach	1.73	**ICD-10**	0VTS0ZZ	Resection of penis, open approach	2.32

© 2025 Balkan E et al. *JAMA Network Open.*

eTable 8. Bivariate Associations between Sociodemographic Factors and Surgery Using a Generalized Estimating Equation for Medicare Beneficiaries Who Are Not Identified as Transgender or Gender Diverse, January 2016-February 2020

	Category	Odds ratio	p-value
Medicare-Medicaid dually enrolled		1.19 (1.02-1.38)	0.03
Age Category Reference Group = 61-65	18-30	1.60 (1.10-2.32)	0.02
	31-40	1.52 (1.06-2.18)	0.02
	41-50	1.45 (1.02-2.07)	0.04
	51-55	0.93 (0.60-1.44)	0.74
	56-60	0.96 (0.62-1.48)	0.85
	66-70	1.00 (0.69-1.46)	0.98
	71-75	0.94 (0.64-1.38)	0.74
	over 75	0.99 (0.70-1.40)	0.94
Race or Ethnicity Reference Group = Black or African American	American Indian or Alaska Native	0.83 (0.55-1.27)	0.39
	Asian or Pacific Islander	0.70 (0.50-0.98)	0.04
	Hispanic	0.78 (0.62-0.99)	0.04
	Non-Hispanic White	0.83 (0.69-1.00)	0.05
	unknown	0.73 (0.50-1.08)	0.21
	Other	0.55 (0.32-0.94)	0.03
Original Reason for Medicare entitlement	age	1.12 (0.89-1.40)	0.33
Reference Group = Disability	ESKD or ESKD and disability*	1.40 (1.10-1.79)	0.006
CMS Region	2: New Jersey, New York, Puerto Rico, Virgin Islands	1.25 (0.89-1.74)	0.19
Reference Group = Region 1 (the Northeast)	3: Delaware, District of Columbia, Maryland, Pennsylvania, Virginia, West Virginia	1.10 (0.78-1.56)	0.59
	4: Alabama, Florida, Georgia, Kentucky, Mississippi, North Carolina, South Carolina, Tennessee	0.91 (0.65-1.29)	0.60
	5: Illinois, Indiana, Michigan, Minnesota, Ohio, Wisconsin	0.96 (0.68-1.36)	0.83
	6: Arkansas, Louisiana, New Mexico, Oklahoma, Texas	0.86 (0.61-1.23)	0.42
	7: Iowa, Kansas, Missouri, Nebraska	0.99 (0.66-1.49)	0.98
	8: Colorado, Montana, North Dakota, South Dakota, Utah, Wyoming	0.95 (0.62-1.45)	0.81
	9: Arizona, California, Hawaii, Nevada, Pacific Territories	0.95 (0.67-1.34)	0.76
	10: Alaska, Idaho, Oregon, Washington	1.15 (0.78-1.69)	0.50
Chronic Conditions: # Reference Group = 0	1	1.01 (0.65-1.56)	0.98
	2-5	1.48 (1.16-1.89)	0.002
	6-9	2.16 (1.69-2.76)	0.000
	10+	4.39 (3.46-5.57)	0.000

© 2025 Balkan E et al. *JAMA Network Open.*

*Medicare's classification of "other" can be interpreted as a non-Hispanic race/ethnicity other than those offered, and "unknown" suggests that the data is missing for an individual.[30]
**ESKD = End Stage Kidney Disease

© 2025 Balkan E et al. *JAMA Network Open.*
