# Supplementary material for: Gender-Affirming Surgery for Transgender and Gender Diverse Medicare Beneficiaries
Source: JAMA Netw Open. 2025 May 1;8(5):e258072. doi: 10.1001/jamanetworkopen.2025.8072 (PMC12046427; doi:10.1001/jamanetworkopen.2025.8072)
Supplement: Supplement 2. — Data Sharing Statement [file jamanetwopen-e258072-s002.pdf]

Data Sharing Statement

Balkan. Gender-Affirming Surgery for Transgender and Gender Diverse Medicare Beneficiaries. *JAMA Netw Open*. Published May 01, 2025. doi:10.1001/jamanetworkopen.2025.8072

Data
Data available: No

Additional Information
Explanation for why data not available: This was a secondary data analysis. No primary data was collected.
